# Focus on the Lymphatic Route to Optimize Drug Delivery in Cardiovascular Medicine

**DOI:** 10.3390/pharmaceutics13081200

**Published:** 2021-08-04

**Authors:** Nolwenn Tessier, Fatma Moawad, Nada Amri, Davide Brambilla, Catherine Martel

**Affiliations:** 1 Department of Medicine, Faculty of Medicine, Université de Montréal, Montreal, QC H3T 1J4, Canada; nolwenn.tessier@icm-mhi.org (N.T.); nada.amri@umontreal.ca (N.A.); 2 Montreal Heart Institute Research Center, Montreal, QC H1T 1C8, Canada; 3 Faculty of Pharmacy, Université de Montréal, Montreal, QC H3T 1J4, Canada; fatma.moawad@umontreal.ca; 4 Department of Pharmaceutics, Faculty of Pharmacy, Beni-Suef University, Beni-Suef 62511, Egypt

**Keywords:** lymphatics, cardiovascular diseases, drug delivery route, nanotechnology

## Abstract

While oral agents have been the gold standard for cardiovascular disease therapy, the new generation of treatments is switching to other administration options that offer reduced dosing frequency and more efficacy. The lymphatic network is a unidirectional and low-pressure vascular system that is responsible for the absorption of interstitial fluids, molecules, and cells from the peripheral tissue, including the skin and the intestines. Targeting the lymphatic route for drug delivery employing traditional or new technologies and drug formulations is exponentially gaining attention in the quest to avoid the hepatic first-pass effect. The present review will give an overview of the current knowledge on the involvement of the lymphatic vessels in drug delivery in the context of cardiovascular disease.

## 1. Introduction

Cardiovascular diseases (CVD) are one of the leading causes of death worldwide [1]. CVD include coronary heart disease, myocardial infarction (MI), heart failure (HF), stroke, and artery diseases [2]. Treatments for cardiovascular diseases are numerous, and the routes of administration are diverse. The chosen drug delivery route is a key determinant of the pharmacodynamics, pharmacokinetics, as well as toxicity of the delivered compounds. Yet, side effects or therapeutic failures are raising concerns, highlighting the need for new administration routes and improved formulation of molecules that reduce their degradation by hepatic metabolism. Drug delivery refers to the methods, approaches, or strategies employed for the transport of pharmaceutical compounds to an organism to achieve a desired therapeutic outcome. With this intent, various routes of administration are used to manage CVD and their risk factors, including parenteral (intravenous (IV), intradermal (ID), intramuscular (IM), subcutaneous (SC), and intraperitoneal (IP)), and transmucosal (oral, nasal, pulmonary, ocular, and genital) and transdermal route [3]. Drug absorption and transport through the lymphatic system makes it possible to avoid hepatic metabolism and is a privileged target in pathologies, such as particular types of cancer (chemotherapeutics [4]) or vaccines [5,6] (HIV [7]), but also for macromolecules [8], and the extensively hepatic-metabolized compounds [9,10].

Discovered in the 17th century [11], the lymphatic system is composed of lymphatic vessels (LV), lymph nodes (LN), and lymphoid organs. LV are organized as initial lymphatics, also called capillaries, localized in the interstitium (i.e., such as the skin, the intestines, and peripheral tissues), pre-collecting and collecting LV. Lymphatic capillaries take up the interstitial fluid that normally consists of immune cells, cellular debris, proteins, electrolytes, chylomicrons, and high-density lipoproteins (HDL) [12,13]. Then, lymph circulates in a unidirectional way due to the presence of valves and lymphatic muscle cells (LMC) surrounding a lymphatic endothelial cell (LEC) monolayer. Lymph passes through the LN and the efferent LV until it reaches the thoracic lymphatic duct and, finally, the bloodstream via the subclavian vein [12]. Given its role in the absorption of interstitial fluids, molecules, and cells from the peripheral tissue, a dual interplay of the lymphatic system in CVD was first brought up in the early 1980s [14] and has gained growing attention in the past few decades [13,15,16,17,18,19,20,21,22]. Atherosclerosis, the main cause of CVD, is characterized by lipid and immune cell accumulation within the artery wall [23]. LV present in the blood vessel walls are involved in the removal of cholesterol from the arterial intima [15]. Further, after an MI, the lymphatic system is involved in the reduction of edema, improvement of cardiac function, and reduction of hypertrophy and fibrosis [24,25,26,27]. Whereas there is an interest in improving lymphatic function to further control CVD onset and progression, targeting the lymphatic system for cardiovascular drug delivery is a promising strategy to limit CVD outcomes. Preferential uptake into the lymphatics is highly dependent on the route of administration and physicochemical properties of the compounds, including size, molecular weight, surface charge, and lipophilicity [28]. This review focuses on the potential key involvement of the lymphatic system in the optimization of drug delivery in CVD.

## 2. Conventional and Novel Therapies to Treat CVD

Historically, small molecules have been used for the treatment of CVD. However, these molecules improve the symptoms and slow down the disease progression without having an actual regenerative effect on the affected tissues or organs [29]. Thus, the remaining unmet clinical needs necessitated the urgent seek for other potential therapeutic options.

Gene therapy is one of the most promising treatment strategies for CVD [30,31,32,33,34], inherited or acquired, through targeting the causative genes engaged in the induction and progression of the disease. It works through replacing defective genes, silencing overexpressed ones or providing functional copies of specific therapeutic genes, thanks to DNA, RNA (siRNA, microRNA, mRNA), and antisense oligonucleotides (ASO) [35]. Back in the 1950s and 1960s, several attempts were made to directly transfect cells with DNA and RNA. Nevertheless, in vivo studies failed to show a noticeable success. Thus, selecting a suitable vector to deliver gene therapy is as important as selecting the agent itself [36,37]. Generally, vectors can be divided into viral and non-viral. The most commonly used viral vectors are retrovirus (RV), adenovirus (AV), adeno-associated virus (AAV), and lentivirus [38]. The most commonly used non-viral vectors include lipid-based vectors using cationic lipids and polymer-based vectors using cationic polymers [39]. Cationic lipids complex with the genetic materials to form lipoplexes or lipid nanoparticles (LNP), while cationic polymers form polyplexes [40]. In 2012, cardiovascular gene therapy was the third most common application for gene therapy (8.4% of the total gene therapy trials). However, clinically, it is still in the infancy stage, and a lot of effort is yet to be expended to correct the underlying basal molecular mechanisms behind different cardiovascular disorders [41,42].

On the other hand, vaccines, developed to produce a long-term immune response against specific antigens, are considered the most economic life-saving medical intervention so far. Previous studies have demonstrated that antigens are trafficked by immune cells from the administration site through the lymphatic network to activate the residing lymphocytes in the secondary lymphoid tissues to produce antibodies [6], a fundamental step for a successful vaccination process. Vaccination against infectious diseases depends on activating T cells and inducing antibody production, following antigen presentation by antigen presenting cells (APC) to T cells through major histocompatibility complex (MHC) molecules. This binding produces cytotoxic T cells that may trigger an autoimmune response. However, the success of vaccination against self-antigen in life-long diseases, such as hypertension, depends on the ability of the vaccine to produce antibodies without triggering a cytotoxic immune response, for safety issues [43]. For example, vaccination against influenza and pneumococcal infection, a potential risk factor for CVD, could have a protective role in populations with a high risk of CVD, including MI and HF [44,45].

Moreover, conventional orally-administered molecules failed to reach satisfaction owing to their poor aqueous solubility, lack of specificity, short half-life, and, hence, low therapeutic outcome and high systemic adverse events [46]. Driven by these facts, researchers have made great efforts to deliver these molecules in a more effective and safer way. One of the most important strategies used for this purpose is nanotechnology. Indeed, using nanotechnology in the chronic management of CVD could revolutionize the cardiovascular healthcare sector. Nanotechnology can serve as a drug delivery platform to improve the characteristics of the free drugs, e.g., solubility, stability, biodistribution, pharmacokinetics, and toxicity profile. Hence, the choice of a suitable carrier has a great impact on the therapeutic outcome [47].

Related to their administration route, the use of conventional cardiovascular therapy, gene-based therapy, vaccines, and nanotechnology to treat CVD is discussed in this review.

## 3. Treating CVD through Various Administration Routes

The following sections describe several routes of administration, with an overview on the lymphatic involvement and several conventional and novel therapies used to treat CVD (Table 1, Table 2, Table 3, Table 4, Table 5 and Table 6).

### 3.1. Oral Administration

Among the various routes of administration, the oral route is the most commonly employed. It exhibits many advantages, including pain avoidance, ease of administration, patient compliance, reduced care cost, and low incidence of cross-infection. Furthermore, it is amenable to various types and forms of pharmaceuticals [48] (Table 1). While some drugs are intended to target the gastrointestinal tract (GIT), the majority are employed to exert a systemic therapeutic effect. Nevertheless, the oral bioavailability of most pharmaceutical compounds depends mainly on their solubility, permeability, and stability in the GIT environment [49,50,51].

Orally-administered compounds enter the systemic circulation through either the portal vein or the intestinal lymphatics after being absorbed, respectively, by the blood or lymphatic vasculature, draining the interstitium. However, these compounds should initially be able to pass through the intestinal epithelium and reach the interstitium to be amenable for transport [10]. Once in the interstitium, two essential factors determine the predominant way of transport: size and lipophilicity. Small molecules/particles are predominantly transported by the blood due to the higher portal blood flow rate as compared to the intestinal lymph flow rate, although they have relatively free access to both blood and lymphatic capillaries. Conversely, large (>500 g/mole) [52] and highly lipophilic molecules (log P > 5, long-chain triglyceride (TG) solubility > 50 mg/g) and particles (up to 10 µm) [53] preferentially enter the highly permeable lymphatics to different extents [10,53,54]. Thus, in order to avoid the hepatic metabolism, the use of a lipophilic molecule is essential, allowing the drug to reach the mesenteric lymphatic vessels permitting the absorption of lipids [55]. The lymphatic system in the intestines is composed of capillaries in the microvilli (lacteals) and collecting lymphatic vessels in the mesentery [56].

- **Diabetes**

Cardiovascular diseases are known as the leading cause of mortality in diabetes mellitus (DM) [57], a chronic debilitating metabolic disorder that spreads worldwide. As presented in Table 1, diabetes is commonly treated by conventional therapy (i.e., metformin, sulfonylureas), while gene therapy, vaccines, and nanotechnology are under intensive investigation [35,58,59,60,61,62,63,64,65,66].

Nanotechnology has attracted an increasing interest in the treatment and management of diabetes mellitus. Oral insulin delivery as a convenient and pain-free alternative to SC injection has been the focus of researchers over the last century [67]. Nanocarriers can be used to protect insulin and other hypoglycemic medications from enzymatic degradation [35]. Indeed, encapsulating insulin in nanoparticles in different studies has shown improved oral bioavailability [68,69,70]. However, the clinical translation of these studies has been so far negative [64]. Several approaches have been employed to improve the encapsulated insulin bioavailability through enhancing its absorption from the intestinal lumen. One of them is via promoting its lymphatic absorption. Indeed, complexing insulin with cationic liposomes has shown a gradual increase in insulin concentration in the lymphatic system over time, upon investigating the absorption pathway in lymph fistula- rat model, indicating a major involvement of the lymphatic system in the transport of these nanoparticles [64]. Similarly, lymphatic transport of insulin-loaded poly lactic-co-glycolic acid (PLGA) nanoparticles was believed to be the reason, in part, beyond its improved bioavailability and sustained hypoglycemic effect [65]. The same approach was used to improve the oral bioavailability of exenatide, the glucagon-like peptide-1 (GLP-1) analogue that is primarily administered SC. Exenatide is injected frequently in high doses to overcome its short plasma half-life. Hence, oral delivery seems a favorable alternative; however, it limited by the poor bioavailability of the peptide drug. Nevertheless, using a phase-changeable nanoemulsion with medium-chain fatty acid has increased the relative bioavailability of exenatide by enhancing its intestinal absorption and lymphatic transport, bypassing the hepatic first-pass effect. Lymphatic involvement in exenatide transport was confirmed by inhibiting the lymphatic pathway using cycloheximide. Results confirmed the absence of nanoparticles in epithelial villi, Peyer’s patches, and major organs [66].

- **Hypertension**

Hypertension is a major risk factor for several CVD. Conventional antihypertensive medications (Table 1) mostly suffer from certain challenges that limit their therapeutic outcome, including short half-life, poor water solubility, low bioavailability, and serious side effects, such as angioedema, bronchospasm, asthma, male breast hyperplasia, reflex tachycardia, and others [46,71,72,73,74,75,76,77,78,79,80,81,82,83,84,85,86,87]. Formulating drugs, such as carvedilol, into nanoemulsion demonstrated a significant improvement in its absorption, permeability, and bioavailability following oral administration [88,89,90,91,92]. The enhanced bioavailability of these medications in nanoemulsion was attributed to their transport via lymphatics, in accordance with the previously established role of nanoemulsion in promoting lymphatic absorption of lipophilic molecules [88,89]. Besides nanoemulsion, other nanocarriers have been widely tested to improve the characteristics of the antihypertensive medications. For instance, felodipine-loaded PLGA were able to bypass the hepatic metabolism by absorption via M-cells of the Peyer’s patches and transport through the lymphatics, facilitated by the negative charges on the particles [93]. Other nanosystems that improved the oral bioavailability and efficacy of antihypertensive medications through promoting lymphatic uptake in preclinical studies include: solid lipid nanoparticles [94], nanostructured lipid carriers [95], proliposomes [96], and Eudragit nanoparticles [97].

- **Hypercholesterolemia and hyperlipidemia**

Hyperlipidemia is a family of disorders characterized by elevated level of lipids in the blood and is a major risk factor for atherosclerosis and CVD. One of the most common hyperlipidemia is hypercholesterolemia [98].

Several medications intended for the treatment of hyperlipidemia and hypercholesterolemia have various constraints that affect their efficacy [99,100]. Nanoparticles promoting lymphatic uptake seems a good strategy to resolve these constraints. For instance, atorvastatin, the widely prescribed statin for the treatment of hyperlipidemia and hypercholesterolemia, has been formulated into several polymeric and lipid-based nanocarriers that enhanced its lymphatic absorption and its oral bioavailability, in consequence (Table 1) [101,102,103,104,105].

The same strategy has been used with other statins to improve their bioavailability through enhancing lymphatic uptake, such as simvastatin [106], rosuvastatin [107], and fluvastatin [108], as well as other agents, such as fibrates [109,110,111] and the cholesterol absorption inhibitor ezetimibe [112,113,114].

**Table 1:** Oral delivery of various treatments for CVD.

Condition	Intervention and Identifier	Target	Dose and Outcome
Diabetes	Metformin		From 500 to 850 mg, 2–3 times a day, during the meal [58]
Diabetes	Sulfonylureas Meglitinide		Dosage is very different from one class of medication to another [59]
Diabetes	Acarbose, Miglitol Voglibose	Carbohydrate digesting enzymes in the brush border	50 mg three times daily (up to 100 mg) [60]
Diabetes	Rosiglitazone Pioglitazone	PPAR-α	Rosiglitazone: 4 mg per day (up to 8 mg) Pioglitazone: 15–30 mg per day [61]
Diabetes	Sitaglipin Vildaglipin Saxaglipin Linaglipin Aloglipin	DPP4	2.5–100 mg once daily depending on the inhibitor used [62]
Diabetes	Dapagliflozin Canagliflozin Empagliflozin	SGLTP2	Dapagliflozin: 2.5–10 mg daily Canagliflozin: 100–300 mg Empagliflozin: 5–25 mg daily [63]
Diabetes	AG019 (NCT03751007) or in combination with the anti-CD3 monoclonal antibody teplizumab		2 or 6 capsules per day for 8 weeks (repeated dose) or for one day (single dose)
Diabetes	Insulin nanocarriers		Protection of insulin from enzymatic degradation Enhancement of stability, intestinal permeability, and bioavailability [35]
Diabetes	Electrostatically-complexed insulin with partially uncapped cationic liposomes		Improved insulin pharmacokinetic profile [64]
Diabetes	Insulin-loaded PLGA		Improved bioavailability and sustained hypoglycemic effect [65]
Diabetes	Exenatide combined to phase-changeable nanoemulsion with medium-chain fatty acid		Enhancement of intestinal absorption and lymphatic transport [66]
HTN	Prazosine Terazosine Doxazosine	Alpha-adrenergic receptor	Prazosine: 3–7.5 mg per day in two doses Terazosine: 1–9 mg per day in the evening at bedtime Doxazosine: 4 mg per day [71]
HTN	Clonidine Methyldopa	Alpha-adrenergic receptor (agonists)	Clonidine: 0.1 mg twice daily [72] Methydopa: 250 mg two to three times daily [73]
HTN	Carvedilol into nanoemulsion	Beta-adrenergic receptors	Significant improvement in its absorption, permeability, and bioavailability [88,89]
HTN	Valsartan, Ramipril and Amlodipine into nanoemulsion		Enhanced solubility, oral bioavailability, and pharmacological outcome [90]
HTN	Felodipine-loaded PLGA nanoparticles	Calcium-channel	Sustained drug release both in vitro and ex vivo [93]
MI HF HTN Arrhythmia	ß-blocker	Beta-adrenergic receptors	Acebutol: 200 mg twice daily [74]
MI HF HTN	Conversion enzyme inhibitors	Conversion enzyme	Captopril: 100 mg per day [75]
MI HF HTN	Valsartan Losartan	Angiotensin II	20 mg twice a day, up to 160 mg [76]
HF HTN	Hydrochlorothiazide Bumetanide	Angiotensin/neprilysin receptor	49 mg/51 mg twice daily and doubled after 2–4 weeks [77]
HF HTN	Sacubitril Valsartan	Calcium channel	5–10 mg daily [78] 60 mg three times daily [79]
HTN Arrhythmia	Amlodipine Diltiazem	Calcium channel	5–10 mg daily [78] 60 mg three times daily [79]
HF	Ivabradine		Bradycardic 5–7.5 mg twice a day [80]
HF MI	Eplerenone Spironolactone	Aldosterone	50 mg once a day [81] and 12.5–25 mg with each intake [82]
HF Arrhythmia	Digoxin		0.25 mg once daily [83]
HF MI HCL	Statin	HMG-CoA	10 mg once daily [84]
MI	Aspirin	Platelets	325 mg, then 81 mg per day [85]
MI	Clopidogrel Prasugrel Ticagrelor	Platelets	300 mg, then 75 mg daily with aspirin 60 mg, then 10 mg daily 180 mg, then 90 mg twice a day [86,87]
HCL	Ezetimibe	Intestinal cholesterol absorption	10 mg once daily [99]
HLD	Tricor Triglide		Fenofibrates 100–300 mg per day [100]
HCL HLD	Atorvastatin formulated into ethylcellulose nanoparticles		Enhanced atorvastatin’s lymphatic absorption and oral bioavailability [101]
HCL HLD	Atorvastatin formulated into nanocrystals prepared with poloxamer 188		Improved atorvastatin’s gastric solubility and bioavailability [102] Reduced circulating cholesterol, TG and LDL
HCL HLD	Atorvastatin formulated into polycaprolactone nanoparticles		Enhanced atorvastatin’s bioavailability [103]
HCL HLD	Nanostructured lipid carriers		Enhanced atorvastatin bioavailability by 2.1 fold compared to the commercial product: lipitor^®^ Reduced the serum level of cholesterol, TG and LDL [104]
HCL HLD	Nanoemulsion		Increased the bioavailability of atorvastatin compared to the commercial tablet ozovas^TM^ [105]
HCL HLD	Simvastatin Rosuvastatin Fluvastatin Fibrates Ezetimibe lipid-based nanoparticles		Improved bioavailability via lymphatic uptake [106,107,108,109,110,111,112,113,114]

PPAR- α: peroxisome proliferator-activated receptor- α; DPP4: dipeptidyl peptidase-4; SGLTP2: Sodium glucose co-transporter-2; PLGA: Poly lactic-co-glycolic acid; HTN: Hypertension; MI: Myocardial infarction; HF: Heart failure; HCL: Hypercholesterolemia; HMG-CoA reductase: Hydroxymethyl glutaryl coenzyme A reductase; HLD: Hyperlipidemia; TG: Triglycerides; LDL: Low density lipoprotein.

### 3.2. Subcutaneous Injection

Subcutaneous injections consist of injecting a molecule under the dermis, in the SC cell layer (interstitial space), and slightly before the muscle, mostly in the abdomen or thigh. The injected molecules will, therefore, either be degraded or phagocytized at the site of injection and join the lymphatic system or the bloodstream [115]. To target the lymphatic system exclusively, this type of injection must be combined with the use of macromolecules. As described in Table 2, subcutaneous injections are used as treatment for various conditions [116,117,118,119,120,121,122,123,124,125,126,127,128,129,130,131,132,133,134,135,136,137,138,139,140,141,142,143].

**Table 2:** Therapies targeting CVD using subcutaneous injection.

Condition	Intervention and Identifier	Therapy	Target	Stage and Status	Dose and Outcome
Diabetes	Insulin				Different types of insulin At least 3 injections per day Dosage adapted to the patient [116]
Diabetes	Exenatide Lixisenatide Liraglutide Exenatide LAR Albiglutide Dulaglutide				GLP-1 analogues [117] Exenatide: 5–10 µg twice a day Lixisenatide: 10–20 µg once daily Liraglutide: 0.6–1.8 mg once daily Exenatide LAR: 2 mg once a week Albiglutide: 30–50 mg once a week Dulaglutide: 0.75–1.5 mg once a week
Diabetes	Vaccine formed of virus-like particles coupled to IAPP		Against the insoluble IAPP- derived amyloid aggregates		Three doses—10 µg Strong immune response against these aggregates and restored insulin production Diminished the amyloid deposits in the pancreatic islets, reduced the level of the pro-inflammatory cytokine IL-1β, and reprieved the onset of amyloid-induced hyperglycemia [118]
Diabetes	IL-1β epitope peptide		Against IL-1β		Three doses—50 µg Enhancement glucose tolerance, improved insulin sensitivity, restored β-cell mass, reduced β-cell apoptosis, and enhanced β-cell proliferation, as well as downregulation of IL-1β expression and inhibition of the inflammatory activity [119,120]
Diabetes	hIL1bQb vaccine (NCT00924105)		Against IL-1β		Six doses—300 µg Mediated a dose-dependent IL-1β-specific antibody response More studies are required to precisely investigate the clinical efficiency of this vaccine [121]
Diabetes	Neutralizing antibodies against DPP4		The GLP-1 and GIP inhibitor, DPP4		Five doses—2–20 µg Increased pancreatic and plasma insulin level and improved postprandial blood glucose level [122]
HTN	hR32 vaccine		Renin-derived peptide		Five doses—500 µg Reduced systolic blood pressure by 15 mmHg [123]
HTN	Angiotensin I vaccine (PMD3117)				Three or four doses—100 µg The vaccine failed to reduce the blood pressure [124]
HTN	AngI-R vaccine		Modifiedendogenous angiotensin I peptide		Four doses—50 µg 15 mmHg reduction in systolic blood pressure and reduced angiotensin I/II level [125]
HTN	ATRQβ-001		Angiotensin II type I receptors		Two doses—100 µg Protective role against target organ damage induced by hypertension [126]
HTN	ATR12181 vaccine		Angiotensin II type I receptors		Nine doses—0.1 mg Attenuated the development of hemodynamic alterations of hypertension [127]
HTN	CYT006-AngQb vaccine		Against angiotensin II		100 or 300 µg Reduction in blood pressure and reduced ambulatory daytime blood pressure [128]
HF HTN	Ang II-KLH vaccine		Angiotensin II		Three doses—5 µg Suppressed post-MI cardiac remodeling and improved cardiac function [129]
MI	Celecoxib loaded in nanoparticles				Promoted vascularization in the ischemic myocardium and delayed HF progression [130]
MI	Chitosan-hyaluronic acid based hydrogel containing deferoxamine-PLGA nanoparticles				Persistent neovascularization in mice [131]
HCL	Alirocumab Evolocumab		PCSK9		One dose every two weeks [132,133]
HCL	Inclisiran		PCSK9		Two doses per year [134]
HoFH HeFH severe HCL	Mipomersen (NCT00607373) (NCT00706849) (NCT00770146) (NCT00794664)	ASO	ApoB	Approved	200 mg once/week. Phase III: reduction in LDL-C [135]
ASCVD HCL HeFH	Inclisiran (NCT03399370) (NCT03400800) (NCT03397121)	siRNA	PCSK9	Approved	284 mg inclisiran, injected on day 1, day 90 and then twice/year Phase III: reduction in LDL-C level [134,136]
FCS	Volanesorsen (NCT02211209)	ASO	ApoC3	Approved	300 mg once/week Phase III: reduction in mean plasma APOC3 and TG level [137]
Elevated LP(a)	ISIS-APO(a)Rx (NCT02160899)	ASO	APO(a)	Phase II (Complete)	Multiple escalating (100–300 mg) doses, injected on a weekly interval for 4 weeks each Phase I/II: reduction in plasma Lp(a) concentration [138]
Elevated LP(a) CVD	AKCEA-APO(a)-LRx (NCT03070782) (NCT02414594) (NCT04023552)	GalNAc3 conjugated-ASO	APO(a)	Phase III (Recruiting)	80 mg administered monthly Phase I/II: reduction in plasma Lp(a) [138]
HTG CVD FCS	AKCEA-APOCIII-LRx (NCT02900027) (NCT03385239) (NCT04568434)	GalNAc3 conjugated-ASO	APOC3	Phase III (Recruiting)	Multiple dosing injected as once/4 weeks for up to 49 weeks Phase II: reduction in ApoC3 and TG levels [139]
HTG FH HLP	Vupanorsen (NCT02709850) (NCT04459767) (NCT04516291)	ASO	ANGPTL3	Phase IIb (Active, Not recruiting)	Multiple escalating dosing (60–160 mg, once/2 or 4 weeks) Phase I: reduction in TG and LDL-C levels [140]
HCL	Neutralizing antibodies against PCSK9		PCSK9		Three doses—5–50 µg Long-lasting reduction in the level of total cholesterol, VLDL and chylomicron [141]
HCL	AT04A		PCSK9		Five doses Strong and persistent anti-PCSK9 antibody production, reduced plasma cholesterol level, attenuated progression of atherosclerosis and reduced vascular and systemic inflammation [142]
HCL	AT04A		PCSK9		Four doses—15 µg and 75 µg Reduced serum LDL-C level and elevated anti-PCSK9 antibody titer [143]
HCL	A peptide representing the mouse ANGPTL3		Angiopoietin-like proteins 3 (ANGPTL3)		Three doses—5 µg Reduced steady-state plasma TGs and promoted LPL activity

GLP-1: glucagon-like peptide-1; IAPP: Islet amyloid polypeptide; DPP4: dipeptidyl peptidase-4; GIP: glucose-dependent insulinotropic polypeptide; HTN: Hypertension; HF: Heart failure; MI: Myocardial infarction; HCL: Hypercholesterolemia; HoFH: Homozygous familial hypercholesterolemia; HeFH: Heterozygous familial hypercholesterolemia; AngII-KLH: Angiotensin II—keyhole-limpet hemocyanin; PCSK9: Proprotein convertase subtilisin/kexin type 9; ASO: Antisense oligonucleotides; ApoB: Apolipoprotein B; LDL-C: low density lipoprotein cholesterol; ASCVD: Atherosclerotic cardiovascular disease; FCS: Familial chylomicronemia syndrome; TG: Triglycerides; LP(a): Lipoprotein(a); APO(a): Apolipoprotein (a); CVD: Cardiovascular diseases; GalNAc3: Triantennary N-acetyl galactosamine; HTG: Hypertriglyceridemia; FH: Familial hypercholesterolemia; HLP: Hyperlipoproteinemia; ANGPTL3: Angiopoietin-like proteins 3; VLDL: Very low density lipoprotein; LPL: Lipoprotein lipase.

- **Diabetes**

The commonly used treatment for diabetes is insulin injections at least three times per day, and the dosage is adapted to the patient [116]. In addition, many vaccines showed significant protection against type 2 diabetes mellitus (T2DM) (Table 2).

Islet amyloid polypeptide (IAPP) is a normally secreted product by β-cells in the pancreas. However, their accumulation in the extracellular space as insoluble aggregates mediates β-cell toxicity and inflammation. Moreover, interleukin 1β (IL-1β) is a key proinflammatory cytokine implicated in pancreatic islets inflammation and insulin resistance in T2DM.

- **Hypertension**

Angiotensinogen (AGT) represents an important target for gene therapy. Indeed, recent studies based on siRNA targeting hepatic AGT, through SC or IV administration, resulted in stable and durable antihypertensive and cardioprotective effect in spontaneously hypertensive rats [144].

On the other hand, three targets in the renin-angiotensin system that have been extensively studied in the preclinical and clinical studies for the treatment of hypertension with vaccines include: renin, angiotensin I, and angiotensin II and its receptors. Renin, as an initiator of the renin-angiotensin system, was the first reported target for vaccination. Furthermore, angiotensin I converts into angiotensin II, which causes dramatic changes in blood pressure. Not only does angiotensin II exert a direct effect on blood pressure, but it also induces the release of the potent vasoconstrictor aldosterone. Direct angiotensin II effect on blood pressure is mediated through stimulating angiotensin II type I receptors that enhance sodium retention and raise blood pressure [145,146]. Thus, blocking the effect of the renin-angiotensin system would have a beneficial role in the management of hypertension.

- **Myocardial infarction**

The high mortality rate associated with MI requires urgent intervention and repair of the affected zones. Advances in this area mainly depend on restoring the blood supply. Angiogenesis is one of the most exciting therapeutic strategies in the management of CVD. Nanotechnology plays an important role in initiating and promoting angiogenesis (or lymphangiogenesis, thanks to VEGF receptor 3), as described in Table 2 [130,131].

- **Hypercholesterolemia and hyperlipidemia**

The use of gene-therapy has also been investigated for the treatment of hypercholesterolemia and hyperlipidemia. Indeed, mipomersen (Kynamro; Kastle Therapeutics) is a second-generation antisense oligonucleotides (ASO) that targets apolipoprotein-B 100 (ApoB-100) and was approved for the treatment of Homozygous familial hypercholesterolemia (HoFH) [147]. It reduces lipoproteins containing the ApoB-100 (e.g., lipoprotein(a) and LDL) through targeting ApoB-100 mRNA in the liver [148]. HoFH is an inherited disorder characterized by low density lipoprotein-cholesterol (LDL-C) uptake and caused by mutations in the *ldl-receptor* (*ldlr*), *apolipoprotein-b* (*apob)*, and *proprotein convertase subtilisin/kexin type 9* (*pcsk9)*. Following SC administration, mipomersen is rapidly absorbed and distributed, compared to a 2 h IV infusion. One of the major accumulation sites of the drug in animals is mesenteric lymph nodes, assuming a considerable absorption by the lymphatics [149]. Kynamro has been withdrawn from the market in 2019 due to safety issues represented in liver toxicity, injection-site reaction, and flu-like symptoms [32,150].

Likewise and for safety issues, represented in thrombocytopenia and bleeding risks, volanesorsen (Waylivra^®^; Akcea Therapeutics, Cambridge, MA, USA) has been denied FDA approval for the treatment of familial chylomicronemia syndrome (FCS), a rare genetic disorder caused mainly by mutations in *lpl* gene or encoding genes required for LPL function [151]. However, in 2019, the European Medicines Agency (EMA) granted it a conditional marketing authorization for genetically confirmed FCS cases with a high risk of pancreatitis and patients with inadequate response to TG-lowering therapy and low-fat diet [152]. FCS is characterized by accumulation of TG-rich lipoproteins in the blood. Apolipoprotein-C3 (ApoC3) plays a regulatory role in the determination of plasma TG level through inhibiting lipoprotein clearance via both LPL-dependent/independent pathways [153]. Thus, inhibiting ApoC3 activity could have a favorable impact on the lipid profile in FCS. Volanesorsen is an ASO that targets and inhibits ApoC3 production in the liver and enhances TG metabolism via the LPL-independent pathway. In rats, one of the main accumulation sites of volanesorsen following SC administration are mesenteric lymph nodes, indicating a reasonable uptake by the lymphatics [154].

Furthermore, inclisiran (Leqvio^®^; Novartis, Schaftenau, Austria), a short-chain, double-stranded siRNA, conjugated to triantennary N-acetylgalactosamine carbohydrates to target the hepatocytes, has been approved in the European Union for the treatment of primary hypercholesterolemia or mixed dyslipidemia in adults, as an adjunct to low-fat diet [155,156]. Inclisiran is injected SC twice a year to produce a long-term anti-hypercholesterolemic effect through targeting PCSK9 [155]. Following SC injection of ^14^C labeled inclisiran in monkeys, lymph nodes showed the third-highest exposure organ, after liver and kidney [157].

On the other hand, several studies reported beneficial effects of active vaccination on hypercholesterolemia. For instance, vaccines targeting angiopoietin-like proteins 3 (ANGPTL3) in mice has reduced the steady-state plasma TGs and promoted LPL activity (Table 2). ANGPTL3 has a key role in TG metabolism and plasma levels through inhibiting LPL activity. People with loss-of-function mutations in ANGPTL3-encoding genes exhibit reduced TG level and a low risk of CVD. Thus, vaccination against ANGPTL3 could serve as a promising strategy for protection against hypertriglyceridemia and relative CVD [158].

### 3.3. Intradermal Injection

Lymphatic capillaries are present in the dermis and, thus, preferentially take up the injected molecules. Unlike the blood capillaries, initial lymphatics lack the basement membrane underlying the endothelial layer. The distal part of initial LV is exclusively composed of LECs with button-like junctions [159], leading to capillaries that have inter-endothelial gaps with size ranges from a few nanometers to several microns [4,160]. Small particles (<10 nm) [4] and medium-sized macromolecules (up to 16 kDa) [161] are mainly transported away from the interstitial spaces by blood capillaries, thanks to mass transport [162,163]. In contrast, lymphatic access of large particles with diameters exceeding 100 nm is hindered by their restricted movement through the interstitium, via diffusion and convection [4]. In between, particles with a size of 10–100 nm [4] and macromolecules with a size of 20–30 kDa [161] show preferential uptake into the highly permeable lymphatic capillaries either passively (paracellular) or actively (transcellular) through the lymphatic endothelial cells [164]. Indeed, it has been shown that the optimal diameter to target the lymphatic vessels in the dermis is 5 to 50 nm in mice [165]. Macromolecules can enter into lymphatics through transcytosis via receptors localized on the surface of LECs, the lymphatic vessel endothelial hyaluronan receptor 1 (LYVE1), and scavenger receptor class B member 1 (SRB1) or p32 [28].

Intradermal therapy, also known as mesotherapy, is used in dermatology and consists of a series of micro-injections of drugs that will slowly diffuse into the peripheral tissues. For cardiovascular diseases or to target the immune system, ID injection would be a privileged pathway to target the lymphatic system. In fact, the skin has a higher lymph flow rate, higher interstitial pressure, and an abundance of APCs (e.g., macrophages, dendritic cells (DCs), and T cells) compared to the other interstitial spaces [166,167]. Then, drugs will be found directly in the lymph and, ultimately, reach the lymph nodes [168]. The antigens present in the lymph will then activate the immune response of T and B lymphocytes [169]. This method is used to treat, for example, alopecia, cystic acne, and psoriasis [170], or for certain vaccines, such as Bacille Calmette-Guérin (BCG) for tuberculosis disease [171].

- **Diabetes**

In type 1 diabetes mellitus (T1DM), the main goal of vaccination is to induce diabetes-specific immune tolerance via vaccination against autoantigens that induce β-cells destruction, through antigen-specific and non-antigen-specific pathways, to restore β cell tolerance and suppress disease progression at early stages [172]. Non-antigen-specific pathway include using antibodies to suppress T cell immune response. However, the limited specificity of this approach can result in serious systemic side effects.

Antigen-specific modulation has proved efficacy in T1DM murine models and, recently, in clinical studies. Vaccination leads to the establishment of a tolerance of effector T cells (Teff) to diabetes-specific autoantigens and induce/expand immunoregulatory T cells (Treg). Teff has an essential role in the development of β cells- specific immune response, while Treg help maintain peripheral tolerance [173,174]. Tolerizing Teff is usually attained via delivery of a high amount of autoantigen to induce exhaustion, clonal anergy or clonal deletion [175]. Proinsulin, among others, is a major B cell autoantigen peptide targeted by CD4^+^ and CD8^+^ Teff at the early stages of the disease. Thus, proinsulin vaccines seem a good option in recently diagnosed cases [176].

Table 3 presents several vaccines used for diabetes through intradermal injection [177,178,179].

**Table 3:** Intradermal administration as treatment for diabetes.

Condition	Intervention and Identifier	Target	Dose and Outcome
Diabetes	Proinsulin peptide vaccine C19-A3	CD4 T cells	Three equal doses—10–100 µg Vaccine was well tolerated [177]
Diabetes	C19-A3 (NCT02837094)	CD4 T cells	Three doses—10 ug In vitro and ex vivo studies of in human skin reported rapid diffusion of the injected particles through the skin layers and preferential uptake by Langerhans cells in the epidermis, which have a primary role in the tolerance mechanism [178]
Diabetes	PIpepTolDC vaccine (NCT04590872)	Tolerogenic DC Vaccine	One dose and another after 28 days No results yet, but, it is believed to be able to produce proinsulin-specific Treg [179]

DC: Dendritic cells; Treg: immunoregulatory T cells.

### 3.4. Intramuscular Injection

Intramuscular injections are used to target the deeper muscle tissue that is highly irrigated. This route of injection allows a rapid absorption and prolonged action. The medication would enter the bloodstream directly and, thus, allow the “bypass” of the hepatic metabolism. It is mainly used for the administration of vaccines [180] (hepatitis, flu virus, tetanus) or with specific pathologies, such as rheumatoid arthritis and multiple sclerosis. It is frequently performed in the upper arm [181] but also in the hip or thigh [182]. It is possible to administer up to 5 mL via this route, based on the site of injection [183]. As lymphatic vessels are present in the skeletal muscle and the connective tissue [184], this leads to the assumption the lymphatic system might be involved in the drug absorption following intramuscular administration. As presented in Table 4, several conditions are treated with this type of injection [185,186,187,188].

**Table 4:** CVD therapies using intramuscular administration.

Condition	Intervention and Identifier	Target	Dose and Outcome
Diabetes	Preproinsulin-encoding plasmid DNA	Pancreatic islets	40% higher survival rate as compared to the control group [185]
HTN	CoVaccine HT (NCT00702221)	Against angiotensin II	Three doses Terminated in 2016 due to dose-limiting adverse effects
HTN	AGMG0201 vaccine	Against angiotensin II	High or low dose (0.2 mg plasmid DNA and 0.5 or 0.25 mg Ang II-KLH conjugate) Ongoing
ACS HF CVD	Inactivated influenza vaccine		Less frequent hospitalization from ACS, hospitalization from HF and stroke [186]
MI	Influenza vaccine		Risk of cardiovascular-related death was significantly lower [187]
CVD MI	Pneumococcal vaccines		Reduced incidence of cardiovascular events and mortality Reduced risk of MI in the elderly [188]
MI HF Stroke	Influenza vaccine (NCT02831608)		The primary endpoints: death, new MI and stent thrombosis Secondary endpoints: patients with hospitalization for HF

HTN: Hypertension; AngII-KLH: Angiotensin II—keyhole-limpet hemocyanin; ACS: Acute coronary syndrome; CVD: cardiovascular disease; HF: Heart failure; MI: Myocardial infarction.

- **Diabetes**

In a study carried out by Abai et al., they found that streptozotocin (STZ)-induced diabetic mice injected IM with preproinsulin-encoding plasmid DNA showed a 40% higher survival rate as compared to the control group. Muscles of the treated group were able to produce insulin, to which the increased survival was attributed [185].

In addition, patients with T2DM could benefit from vaccination against obesity, being a leading risk factor for T2DM. The main targets for obesity vaccines include GIP, adipose tissue antigen, somatostatin, and ghrelin [189]. Other vaccines that showed considerable benefits and are recommended for patients with DM are influenza, pneumococcal, hepatitis A and B, varicella vaccines, and others [190].

- **Heart failure**

The last few decades have witnessed several studies that target the major pathogenic pathways implicated in HF. Among these targets: cardiac muscle contractility, angiogenesis, cytoprotection, and stem cell homing [191]. For instance, the β-adrenergic system plays a major role in the regulation of cardiac contractility. Up-regulation of the myocardial G-protein-coupled receptor kinase 2 (GRK2) protein in failing hearts was found to desensitize and down-regulate β-adrenergic receptors by up to 50% [192], which lead to the hypothesis that cardiac function could be improved with the GRK2-inhibitor peptide beta adrenergic receptor kinase carboxyl-terminus (βARKct). Several studies in mice, rats, rabbits, and pigs showed that overexpressing βARKct helped to prevent the development and progression of HF, restored β-adrenergic receptor sensitization and prolonged the survival [193,194,195,196,197], and improved and sustained contractile function [198].

Furthermore, disrupted calcium (Ca^2+^) homeostasis in the cardiomyocytes was found to be a consistent feature in HF, which is linked to down-regulation in the expression of sarcoplasmic/endoplasmic reticulum Ca^2+^ ATPase 2a (SERCA2a) protein pump and reduced cardiac contractility. The prominent success of SERCA2a gene transfer in enhancing cardiac contractility has paved the way to launch the clinical trial CUPID “Ca^2+^ Upregulation by Percutaneous Administration of Gene Therapy in Cardiac Disease” [199,200]. CUPID was the first gene therapy clinical trial for HF and used AAV1 vectors for SERCA2a gene delivery [201].

- **Hypercholesterolemia and hyperlipidemia**

An AAV1-based therapy encoding LPL gene has been commercialized and approved since 2012 for the treatment of lipoprotein lipase deficiency (LPLD), alipogene tiparvovec (Glybera; uniQure Biopharma) [202]. LPLD, otherwise identified as chylomicronaemia syndrome, is a rare, hereditary autosomal disease resulting from loss-of-function mutations in LPL gene, which has a central role in the breakdown and clearance of triglyceride (TG)-rich lipoproteins (chylomicron and very low-density lipoprotein (VLDL)). As a result, patients with LPLD are prone to severe, recurrent attacks of pancreatitis, and they are at high risk of developing diabetes mellitus [203]. Although the highest level of vector DNA was observed at the injection site following IM injection of the drug in mice, a considerable amount was detected in the draining lymph nodes indicating the involvement of the lymphatic system in drug uptake [204]. Clinical trials reported immediate LPL expression following AAV1 injection and an associated long-term enhancement in chylomicron clearance. In addition, a retrospective study has documented a potential benefit of Glybera in reducing the frequency of pancreatitis episodes [205]. However, in 2017, the company declined Glybera’s marketing authorization renewal due to the unprofitability driven by the disease rarity [206].

### 3.5. Intramyocardial Injection

Direct intramyocardial injection is the most effective and commonly used way for gene delivery to the heart owing to its ability to achieve a high concentration of the injected compound at the injection site [207]. It is a preferential route to directly target lymphatic vessels due to their high density in the myocardium [159,208]. Various CVD and their treatments via intramyocardial injection are presented in Table 5 [201,209,210,211,212,213,214,215,216,217,218,219,220,221,222,223,224].

- **Myocardial infarction**

The use of nanotechnology as treatment demonstrated significant protection against post-reperfusion myocardial injury in ischemic hearts via reducing the elevated level of reactive oxygen species (ROS) and the intracellular Ca^2+^ (Table 5). These nanoparticles protected the cardiomyocytes in isolated hearts from damage and oxidative stress, an approach that could be very useful upon in vivo application [223]. Although two nanoparticle systems have received FDA approval for post-MI imaging, none was approved for therapeutic purposes [225].

On the other hand, modulating one or more of the following growth factors, vascular endothelial growth factor (VEGF) [226], fibroblast growth factor (FGF) [227], hepatocyte growth factor (HGF) [228], and platelet-derived growth factor (PDGF) [229] could have a repairing effect on the affected zones within the myocardium (Table 5).

**Table 5:** Use of intramyocardial injections in several therapies targeting CVD.

Condition	Intervention and Identifier	Therapy	Target	Stage and Status	Dose and Outcome
HF	Ad5.hAC6 (NCT007)	Ad5	AC6	Phase I/II (Completed)	Single administration of escalating doses (3.2 × 10^9^ vp to 10^12^ vp) Phase II: Reduced HF admission rate. Enhanced left ventricular function beyond the optimal HF therapy following a single administration [209]
HF	Ad5.hAC6 (NCT03360448)	Ad5	AC6	Phase III (withdrawn)	Phase III: withdrawn for re-evaluation
HF	MYDICAR (NCT00454818)	AAV1	SERCA2a	Phase I/II (Completed)	Single administration of escalating doses (1.4 × 10^11^–1 × 10^13^ DRP of AAV1/SERCA2a) Phase I/II (CUPID): high-dose treatment resulted in increased time and reduced frequency of cardiovascular events within a year and reduced cardiovascular hospitalizations [210]
HF	MYDICAR (NCT01643330)	AAV1	SERCA2a	Phase IIb (completed)	Single infusion of 1 × 10^13^ DRP of AAV1/SERCA2a Phase IIb (CUPID-2b): no improvement was observed at the tested dose in patients with HF during the follow-up period [201]
HF	MYDICAR (NCT01966887)	AAVI	SERCA2a	Phase II (Terminated)	1 × 10^13^ DRP of AAV1/SERCA2a as a single intracoronary infusion Phase II: no improvement observed in the ventricular remodeling.The study terminated driven by the CUPID-2 trial neutral outcome [211]
HF	SRD-001 (NCT04703842)	AAVI	SERCA2a	Phase I/II (Active, not recruiting)	Single administration of 3 × 10^13^ vg CUPID-3: aims to investigate the safety and efficacy of SRD-001 in anti-AAV1 neutralizing antibody-negative subjects with HFrEF
HF CVD	INXN-4001 (NCT03409627)	Non-viral, triple effector plasmid	SDF-1α, S100A1, VEGF-165	Phase I (Completed)	Single 80 mg dose, given in 40 mL or 80 mL at a rate of 20 mL/min Phase I: an improvement in the quality of life in 50% of patients was reported [212]
HF	ACRX-100 (NCT01082094)	Plasmid DNA	SDF-1	Phase I (Completed)	Single escalating doses, injected at multiple sites Preclinical studies: enhanced vasculogenesis and improved cardiac function reported with all doses [213]
HF	JVS-100 (NCT01643590)	Plasmid DNA	SDF-1	Phase II (Completed)	Single injection of escalating doses (15 and 30 mg) Phase II (STOP-HF): JVS-100 showed potential to improve cardiac function through reducing left ventricular remodeling and improving ejection fraction [214]
HF	JVS-100 (NCT01961726)	Plasmid DNA	SDF-1	Phase I/II (Unknown)	Single injection of escalating doses (30 and 45 mg) Phase I (RETRO-HF): JVS-100 showed promising signs of clinical efficacy [215]
HF	AZD8601 (NCT02935712) (NCT03370887)	mRNA	VEGF-A165	Phase IIa (Active, not recruiting)	Single injection of escalating doses (3 mg and 30 mg) Preclinical studies: promoted angiogenesis, improved cardiac function and enhanced survival were reported [216] Phase I: ID injection of AZD8601 was well tolerated and enhanced the basal skin blood flow [217]
HF	NAN-101 (NCT04179643)	AAV	I-1c	Phase I (Recruiting)	Single escalating doses (3 × 10^13^ vg–3 × 10^14^ vg) of NAN-101 Preclinical studies: enhancement in left ventricular ejection fraction and improved cardiac performance [218]
AMI IHD	VM202RY (NCT01422772) (NCT03404024)	DNA plasmid	HGF-X7	Phase II (Recruiting)	Single escalating (0.5–3 mg) doses, administered into multiple sites Phase I: improved myocardial function and wall thickness [219,220]
MI Angina pectoris	AdVEGF-D (NCT01002430)	AV	VEGF-D	Phase I/IIa (Completed)	Single escalating (1 × 10^9^–1 × 10^11^ Vpu) doses, injected into multiple sites in the endocardium Phase 1/IIa: AdVEGF-D improved myocardial perfusion reserve in the injected region [220]
MI	Ad-HGF (NCT02844283)	AV	HGF	Phase I/II (Unknown)	Single dose Preclinical studies: Ad-HGF preserved cardiac function, reduced infarct size, and improved post-MI cardiac remodeling [221]; fractional repeated dosing significantly improved cardiac function compared with single injection [222]
MI	L-type Ca^2+^ channels’ AID peptide and antioxidant molecule (curcumin) in poly nanoparticles				Reduced the elevated level of ROS and the intracellular Ca^2+^ [223]
LPLD	Alipogene tiparvovec (NCT00891306)	AAV	LPL	Approved	1 × 10^12^ GC/kg Phase II/III: reduction in mean total plasma and chylomicron TG level [224]

HF: Heart failure; hAC6: Human adenylyl cyclase type 6; vp: Virus particles; AAV: Adeno-associated virus; SERCA2a: Sarcoplasmic/endoplasmic reticulum Ca^2+^-ATPase; DRP: DNase-resistant particles; HFrEF: HF with reduced ejection fraction; CVD: Cardiovascular diseases; SDF-1a: stromal cell-derived factor 1; VEGF: Vascular endothelial growth factor; I-1c: Constitutively active inhibitor-1; vg: Viral genomes; AMI: Acute myocardial infarction; IDH: Ischemic heart disease; HGF-X7: Hepatocyte growth factor-X7; AV: Adenovirus; Vpu: Viral protein U; HGF: Hepatocyte growth factor; AID: alpha-interacting domain; ROS: reactive oxygen species; LPL: Lipoprotein lipase; TG: Triglycerides; GC: Genome copies.

### 3.6. Intravenous Injection

Intravenous injections are often used for rehydration, nutrition, and therapeutic treatments (for example, blood transfusion or chemotherapy), as well as to avoid hepatic metabolism [230]. The interest of this route of administration is the continuous treatment, or regular frequencies, by the installation of a catheter [231]. However, the lymphatic system is only scarcely involved following IV injections [232,233,234]. Table 6 presents several conditions treated with this type of injection [74,83,85,235,236,237,238,239,240,241,242,243,244,245,246,247,248].

**Table 6:** Intravenous administration of medication as treatment for CVD.

Condition	Intervention and Identifier	Therapy	Target	Stage and Status	Dose and Outcome
HTN	NO-releasing nanoparticles				Reduction in the mean arterial blood pressure [235]
HF Arrhythmia	Digoxin				Dose: 0.25 mg once daily [83]
MI HF HTN Arrhythmia	ß-blocker		Beta-adrenergic receptors		Acebutol: 200 mg twice daily [74]
HF	Mesoporous silicon vector (Nanoconstruct)				Able to internalize, accumulate, and traffic within the cardiomyocytes [236]
HF	Combination of biocompatible magnetic nanoparticles and low-frequency magnetic stimulation		Cardio-myocytes		Managed the drug release by controlling the applied frequencies [237]
HF	S100A1-loaded nanoparticles, decorated with N-acetylglucosamine				Regulated Ca^2+^ release and restored contractile function in the isolated failing cardiomyocytes [238]
HF	Biodegradable nanoparticles conjugated with myocyte-targeting peptide and PDT-enabling photosensitizer	PDT	Cardio-myocytes		Induced cell-specific death upon application of laser light, leaving adjacent and surrounding cells completely intact [239]
MI	Unfractionated heparin				Anticoagulant 60 IU/kg for initial bolus 12 IU/kg/h for maintenance [240]
MI	Aspirin		Platelets		325 mg, then 81 mg per day [85]
MI	Human recombinant VEGF-165				Significant improvement in the infarcted zone perfusion and cardiac function for up to six weeks post-MI [241].
MI	Nanoparticles containing siRNA				Anti-inflammatory effect in the infarcted heart and reduction of the post-MI heart failure [242]
MI	Magnetic nanoparticles-loaded cells				Robust improvement in the left ventricular and cardiac function [243]
MI	Insulin-like growth factor electrostatically-complexed with PLGA nanoparticles				Higher incidence in preventing cardiomyocytes’ apoptosis, reducing infarct size, and enhancing left ventricular function [244]
MI	Pitavastatin in PLGA nanoparticles				Cardioprotective effect against ischemia-reperfusion injury [245]
HoFH	AAV8.TBG.HldlR (NCT02651675)	AAV	hLDLR	Phase I/II (Completed)	Single dose Preclinical studies: reduction in total cholesterol [246,247]
Elevated LDL-C	ALN-PCS02 (NCT01437059)	siRNA	PCSK9	Phase I (Completed)	Single escalating (15 and 400 μg/kg) doses Phase I: reduction in the level of circulating PCSK9 protein and LDL-C [248]

HTN: Hypertension; NO: nitric oxide; HF: Heart failure; MI: Myocardial infarction; PDT: Photodynamic therapy; VEGF: Vascular endothelial growth factor; PLGA: Poly lactic-co-glycolic acid; AAV: Adeno-associated virus; HoFH: Homozygous familial hypercholesterolemia; hLDLR: Human low density lipoprotein receptor; TBG: Thyroxine-binding globulin; LDL-C: low density lipoprotein cholesterol.

- **Diabetes**

The first successful in vivo gene therapy study was completed in the early 1980s, when Nicolau et al. administered rats IV with liposome encapsulating preproinsulin 1-encoding plasmid DNA and demonstrated a significant hypoglycemic effect as well as elevated insulin level in the blood [249]. Ever since, several studies have been published in an attempt to control diabetes through targeting different tissues, such as pancreas [250], muscle [251,252], liver [253,254,255], and K-enteroendocrine cells (K cells) [256]. For effective hypoglycemic therapy, these tissues should be able to produce or secrete insulin in a glucose-dependent manner [257,258].

As for vaccination, several preclinical [259,260] and clinical [261,262,263] studies have demonstrated the efficacy of IV administration of anti-CD3 monoclonal antibodies in restoring β cell tolerance and reversing diabetes in recent-onset T1DM, via targeting the autoreactive T cells.

- **Hypertension**

Antisense oligonucleotides-mediated inhibition of β1-receptors expression in hypertensive rats has resulted in a durable antihypertensive effect for more than two weeks after a single IV dose [264]. Moreover, Huang et al. showed that targeting renal G protein-coupled receptor kinase type 4 (GRK4) with siRNA helped to reduce the blood pressure significantly in spontaneously hypertensive rats [265].

To the best of our knowledge, none of the gene therapy studies targeting hypertension have reached the clinical trials so far [266].

From another perspective, nanoparticles can be used for controlling blood pressure by the generation/release of the vasodilator molecule nitric oxide (NO). Indeed, Cabrales et al. reported a reduction in the mean arterial blood pressure by using NO-releasing nanoparticles. Upon administration, hydration of these nanoparticles resulted in the release of NO at a therapeutic level into the circulation. NO acts by inducing vasodilatation and promoting microvascular perfusion [235]. In this study, NO-releasing nanoparticles were delivered via IV infusion; however, they could be injected IP or IM [235].

- **Heart failure**

So far, myocardial, intracoronary, and pericardial injections are the most commonly used to transduce the myocardium. IV injection offers a less invasive way; however, the low transduction efficiency limits its application [191]. A nanoconstruct called mesoporous silicon vector was able to internalize, accumulate, and traffic within the cardiomyocytes following IV administration in HF murine model, without significant toxicity. This nanoconstruct could serve as a platform for therapeutic and diagnostic purposes in HF [236]. Indeed, as described in Table 6, the use of nanotechnology in this pathology seems promising.

- **Myocardial infarction**

Management of MI at the early stages is essential to avoid the development of irreversible HF, due to the poor cardiomyocytes turnover (Table 6) [267]. IV-injected human recombinant VEGF-165 (an important angiogenesis factor) has resulted in a significant improvement in the infarcted zone perfusion and cardiac function for up to six weeks post-MI [241]. Furthermore, post-MI extended inflammation is another potential target, being a risk factor for post-MI heart failure. Indeed, a study published in 2013 showed that IV administration of nanoparticles containing siRNA in atherosclerotic mice, with coronary ligation-induced MI, produced a significant anti-inflammatory effect in the infarcted heart and reduction of the post-MI heart failure. This effect reflected an improved inflammatory resolution and reduced monocyte numbers in the infarcted area [242].

On the other hand, liposomes, the most primitive form in nanomedicine, could have a great potential for targeting and accumulation in ischemic tissues, following the less invasive IV administration. This could be achieved through attaching targeting moieties and used for diagnosis or therapy [268]. These targeting nanoparticles were exploited to deliver different therapeutic products, e.g., proangiogenic factors [269], genetic materials [270], and cytokines [271]. Moreover, nanoparticles could be used in cell replacement therapy post-MI to restore cardiac function (Table 6).

### 3.7. Intraperitoneal Injection

Intraperitoneal administration, in which therapeutic compounds are injected directly into the peritoneal cavity, is another attractive approach of the parenteral extravascular strategies. It is used specifically for the local treatment of peritoneal cavity disorders, e.g., peritoneal malignancies and dialysis. The peritoneal cavity contains the abdominal organs and the peritoneal fluid, normally composed of water, proteins, electrolytes, immune cells, and other interstitial fluid substances [272]. The high absorption rate associated to IP administration is promoted by the vast blood supply to the peritoneal cavity, along with its large surface area, which is further increased by the microvilli covering the mesothelial layer [273]. Injected compounds can enter the circulatory system after IP injection via both blood and lymphatic capillaries draining the peritoneal submesothelial layer [273,274,275]. Besides, the peritoneal absorption of molecules is greatly affected by their physicochemical characteristics. This route of administration also allows for the injection of large volumes (up to 10 mL) [273]. Extensive experimental studies carried out on animals have revealed that the peritoneal cavity has favorable absorption of lipophilic and unionized compounds [276]. This type of injection is most exploited for preclinical studies, since it is the simplest to perform, especially in small animals and with little impact on the animals’ stress [273,277]. IP use in humans is limited, despite showing many benefits in previous studies and even being recommended, for certain types of chemotherapy, by the National Cancer Institute [278,279,280].

- **Diabetes**

Cheung et al. were able to produce long-term protection against diabetes by using GIP promoters in transgenic mice treated with STZ to damage their pancreatic β cells [256].

- **Myocardial infarction**

IP injection of methotrexate-loaded lipid core nanoparticle in MI rat model has mediated angiogenesis through enhancing the expression of myocardial VEGF. Importantly, it reduced myocytes’ hypertrophy, necrosis, and infarct size, effects that were not observed with the free methotrexate [281].

## 4. Conclusions

Treatments for cardiovascular diseases are numerous, and the routes of administration are diverse. However, side effects or therapeutic failures are also present. Therefore, improvement in therapeutic delivery is essential. This review highlights new administration routes and improved formulation of molecules that ameliorate their efficacy via lymphatic transport. Thus, ensuring an optimal lymphatic transport throughout the body would not only directly reduce CVD [16,25,164,217,220,282,283] but would also allow a proper drug delivery to the various targeted organs. Taken together, the combination of the use of nanotechnology, vaccination, and gene therapy, along with the appropriate administration route to target the lymphatic system, thus, seems to be a promising target for the prevention and treatment of cardiovascular diseases.

## Data Availability

Not applicable.

## References

[1] Kaptoge S., Pennells L., De Bacquer D., Cooney M.T., Kavousi M., Stevens G., Riley L.M., Savin S., Khan T., Altay S. (2019). World Health Organization cardiovascular disease risk charts: Revised models to estimate risk in 21 global regions. Lancet Glob. Health.

[2] Virani S.S., Alonso A., Aparicio H.J., Benjamin E.J., Bittencourt M.S., Callaway C.W., Carson A.P., Chamberlain A.M., Cheng S., Delling F.N. (2021). Heart disease and stroke statistics—2021 update: A report from the American Heart Association. Circulation.

[3] Tiwari G., Tiwari R., Sriwastawa B., Bhati L., Pandey S., Pandey P., Bannerjee S.K. (2012). Drug delivery systems: An updated review. Int. J. Pharm. Investig..

[4] Ryan G.M., Kaminskas L.M., Porter C.J. (2014). Nano-chemotherapeutics: Maximising lymphatic drug exposure to improve the treatment of lymph-metastatic cancers. J. Control. Release.

[5] Maisel K., Sasso M.S., Potin L., Swartz M.A. (2017). Exploiting lymphatic vessels for immunomodulation: Rationale, opportunities, and challenges. Adv. Drug Deliv. Rev..

[6] Pal I., Ramsey J.D. (2011). The role of the lymphatic system in vaccine trafficking and immune response. Adv. Drug Deliv. Rev..

[7] Sleeman J.P. (2006). The relationship between tumors and the lymphatics: What more is there to know?. Lymphology.

[8] Porter C.J., Charman S.A. (2000). Lymphatic transport of proteins after subcutaneous administration. J. Pharm. Sci..

[9] Zhang X.-Y., Lu W.-Y. (2014). Recent advances in lymphatic targeted drug delivery system for tumor metastasis. Cancer Biol. Med..

[10] Yáñez J.A., Wang S.W., Knemeyer I.W., Wirth M.A., Alton K.B. (2011). Intestinal lymphatic transport for drug delivery. Adv. Drug Deliv. Rev..

[11] Asellius G. (1627). De Lactibus Sive Lacteis Venis.

[12] Cueni L.N., Detmar M. (2008). The lymphatic system in health and disease. Lymphat. Res. Biol..

[13] Milasan A., Farhat M., Martel C. (2020). Extracellular Vesicles as Potential Prognostic Markers of Lymphatic Dysfunction. Front. Physiol..

[14] Lemole G.M. (1981). The role of lymphstasis in atherogenesis. Ann. Thorac. Surg..

[15] Martel C., Li W., Fulp B., Platt A.M., Gautier E.L., Westerterp M., Bittman R., Tall A.R., Chen S.-H., Thomas M.J. (2013). Lymphatic vasculature mediates macrophage reverse cholesterol transport in mice. J. Clin. Investig..

[16] Milasan A., Smaani A., Martel C. (2019). Early rescue of lymphatic function limits atherosclerosis progression in Ldlr−/− mice. Atherosclerosis.

[17] Yeo K.P., Lim H.Y., Thiam C.H., Azhar S.H., Tan C., Tang Y., See W.Q., Koh X.H., Zhao M.H., Phua M.L. (2020). Efficient aortic lymphatic drainage is necessary for atherosclerosis regression induced by ezetimibe. Sci. Adv..

[18] Singla B., Lin H.P., Chen A., Ahn W., Ghoshal P., Cherian-Shaw M., White J., Stansfield B.K., Csányi G. (2021). Role of R-spondin 2 in arterial lymphangiogenesis and atherosclerosis. Cardiovasc. Res..

[19] Milasan A., Jean G., Dallaire F., Tardif J.C., Merhi Y., Sorci-Thomas M., Martel C. (2017). Apolipoprotein A-I Modulates Atherosclerosis Through Lymphatic Vessel-Dependent Mechanisms in Mice. J. Am. Heart Assoc..

[20] Milasan A., Ledoux J., Martel C. (2015). Lymphatic network in atherosclerosis: The underestimated path. Future Sci. OA.

[21] Milasan A., Tessandier N., Tan S., Brisson A., Boilard E., Martel C. (2016). Extracellular vesicles are present in mouse lymph and their level differs in atherosclerosis. J. Extracell. Vesicles.

[22] Milasan A., Dallaire F., Mayer G., Martel C. (2016). Effects of LDL Receptor Modulation on Lymphatic Function. Sci. Rep..

[23] Libby P., Ridker P.M., Hansson G.K. (2011). Progress and challenges in translating the biology of atherosclerosis. Nature.

[24] Henri O., Pouehe C., Houssari M., Galas L., Nicol L., Edwards-Lévy F., Henry J.-P., Dumesnil A., Boukhalfa I., Banquet S. (2016). Selective Stimulation of Cardiac Lymphangiogenesis Reduces Myocardial Edema and Fibrosis Leading to Improved Cardiac Function Following Myocardial Infarction. Circulation.

[25] Klotz L., Norman S., Vieira J.M., Masters M., Rohling M., Dubé K.N., Bollini S., Matsuzaki F., Carr C.A., Riley P.R. (2015). Cardiac lymphatics are heterogeneous in origin and respond to injury. Nature.

[26] Vieira J.M., Norman S., Del Campo C.V., Cahill T.J., Barnette D.N., Gunadasa-Rohling M., Johnson L.A., Greaves D.R., Carr C.A., Jackson D.G. (2018). The cardiac lymphatic system stimulates resolution of inflammation following myocardial infarction. J. Clin. Investig..

[27] Vuorio T., Ylä-Herttuala E., Laakkonen J.P., Laidinen S., Liimatainen T., Ylä-Herttuala S. (2018). Downregulation of VEGFR3 signaling alters cardiac lymphatic vessel organization and leads to a higher mortality after acute myocardial infarction. Sci. Rep..

[28] Trevaskis N.L., Kaminskas L.M., Porter C.J.H. (2015). From sewer to saviour—targeting the lymphatic system to promote drug exposure and activity. Nat. Rev. Drug Discov..

[29] Li T., Liang W., Xiao X., Qian Y.J. (2018). Nanotechnology, an alternative with promising prospects and advantages for the treatment of cardiovascular diseases. Int. J. Nanomed..

[30] Wong M.S., Hawthorne W.J., Manolios N. (2010). Gene therapy in diabetes. Self Nonself.

[31] Phillips M.I. (2001). Gene therapy for hypertension: Sense and antisense strategies. Expert Opin. Biol. Ther..

[32] Tromp T.R., Stroes E.S., Hovingh G.K. (2020). Gene-based therapy in lipid management: The winding road from promise to practice. Expert Opin. Investig. Drugs.

[33] Kieserman J.M., Myers V.D., Dubey P., Cheung J.Y., Feldman A.M. (2019). Current landscape of heart failure gene therapy. J. Am. Heart Assoc..

[34] Shimamura M., Nakagami H., Taniyama Y., Morishita R. (2014). Gene therapy for peripheral arterial disease. Expert Opin. Biol. Ther..

[35] Zhao R., Lu Z., Yang J., Zhang L., Li Y., Zhang X. (2020). Drug Delivery System in the Treatment of Diabetes Mellitus. Front. Bioeng. Biotechnol..

[36] Avery O.T., MacLeod C.M., McCarty M. (1944). Studies on the chemical nature of the substance inducing transformation of pneumococcal types: Induction of transformation by a desoxyribonucleic acid fraction isolated from pneumococcus type III. J. Exp. Med..

[37] Meyerson S.L., Skelly C.L., Curi M.A., Schwartz L.B. (2000). Gene therapy for cardiovascular disease. Semin. Cardiothorac. Vasc. Anesth..

[38] Bulcha J.T., Wang Y., Ma H., Tai P.W., Gao G. (2021). Viral vector platforms within the gene therapy landscape. Signal Transduct. Target. Ther..

[39] Su C.-H., Wu Y.-J., Wang H.-H., Yeh H.-I. (2012). Nonviral gene therapy targeting cardiovascular system. Am. J. Physiol. Heart Circ. Physiol..

[40] Hall A., Lächelt U., Bartek J., Wagner E., Moghimi S.M. (2017). Polyplex Evolution: Understanding Biology, Optimizing Performance. Mol. Ther..

[41] Scimia M.C., Gumpert A.M., Koch W.J. (2014). Cardiovascular gene therapy for myocardial infarction. Expert Opin. Biol. Ther..

[42] Cannatà A., Ali H., Sinagra G., Giacca M. (2020). Gene therapy for the heart lessons learned and future perspectives. Circ. Res..

[43] Nakagami H., Morishita R. (2018). Recent advances in therapeutic vaccines to treat hypertension. Hypertension.

[44] Siriwardena A.N. (2012). Increasing Evidence That Influenza Is a Trigger for Cardiovascular Disease.

[45] Singanayagam A., Elder D., Chalmers J.D. (2012). Is community-acquired pneumonia an independent risk factor for cardiovascular disease?. Eur. Respir. J..

[46] Deng Y., Zhang X., Shen H., He Q., Wu Z., Liao W., Yuan M. (2019). Application of the Nano-Drug Delivery System in Treatment of Cardiovascular Diseases. Front. Bioeng. Biotechnol..

[47] Sezer A.D. (2014). Application of Nanotechnology in Drug Delivery.

[48] Zhang J., Xie Z., Zhang N., Zhong J. (2017). Nanosuspension drug delivery system: Preparation, characterization, postproduction processing, dosage form, and application. Nanostructures for Drug Delivery.

[49] Fox C.B., Kim J., Le L.V., Nemeth C.L., Chirra H.D., Desai T.A. (2015). Micro/nanofabricated platforms for oral drug delivery. J. Control. Release.

[50] Trevaskis N.L., McEvoy C.L., McIntosh M.P., Edwards G.A., Shanker R.M., Charman W.N., Porter C.J. (2010). The role of the intestinal lymphatics in the absorption of two highly lipophilic cholesterol ester transfer protein inhibitors (CP524,515 and CP532,623). Pharm. Res..

[51] Vinarov Z., Abdallah M., Agundez J.A.G., Allegaert K., Basit A.W., Braeckmans M., Ceulemans J., Corsetti M., Griffin B.T., Grimm M. (2021). Impact of gastrointestinal tract variability on oral drug absorption and pharmacokinetics: An UNGAP review. Eur. J. Pharm. Sci..

[52] Brocks D.R., Davies N.M. (2018). Lymphatic drug absorption via the enterocytes: Pharmacokinetic simulation, modeling, and considerations for optimal drug development. J. Pharm. Pharm. Sci..

[53] Jenkins P., Howard K., Blackball N., Thomas N., Davis S., O’hagan D.T. (1994). Microparticulate absorption from the rat intestine. J. Control. Release.

[54] Charman W., Stella V.J. (1986). Estimating the maximal potential for intestinal lymphatic transport of lipophilic drug molecules. Int. J. Pharm..

[55] Cifarelli V., Eichmann A. (2019). The Intestinal Lymphatic System: Functions and Metabolic Implications. Cell. Mol. Gastroenterol. Hepatol..

[56] Miller M.J., Newberry R.D. (2010). Microanatomy of the intestinal lymphatic system. Ann. N. Y. Acad. Sci..

[57] Baena-Díez J.M., Peñafiel J., Subirana I., Ramos R., Elosua R., Marín-Ibañez A., Guembe M.J., Rigo F., Tormo-Díaz M.J., Moreno-Iribas C. (2016). Risk of Cause-Specific Death in Individuals With Diabetes: A Competing Risks Analysis. Diabetes Care.

[58] Sanchez-Rangel E., Inzucchi S.E. (2017). Metformin: Clinical use in type 2 diabetes. Diabetologia.

[59] Sola D., Rossi L., Schianca G.P.C., Maffioli P., Bigliocca M., Mella R., Corlianò F., Fra G.P., Bartoli E., Derosa G. (2015). Sulfonylureas and their use in clinical practice. Arch. Med. Sci..

[60] van de Laar F.A. (2008). Alpha-glucosidase inhibitors in the early treatment of type 2 diabetes. Vasc. Health Risk Manag..

[61] Lebovitz H.E. (2019). Thiazolidinediones: The Forgotten Diabetes Medications. Curr. Diabetes Rep..

[62] Gallwitz B. (2019). Clinical Use of DPP-4 Inhibitors. Front. Endocrinol..

[63] Neuen B.L., Cherney D.Z., Jardine M.J., Perkovic V. (2019). Sodium-glucose cotransporter inhibitors in type 2 diabetes: Thinking beyond glucose lowering. CMAJ.

[64] Kim K.S., Kwag D.S., Hwang H.S., Lee E.S., Bae Y.H. (2018). Immense insulin intestinal uptake and lymphatic transport using bile acid conjugated partially uncapped liposome. Mol. Pharm..

[65] Jain S., Rathi V.V., Jain A.K., Das M., Godugu C. (2012). Folate-decorated PLGA nanoparticles as a rationally designed vehicle for the oral delivery of insulin. Nanomedicine.

[66] Lin P.Y., Chen K.H., Miao Y.B., Chen H.L., Lin K.J., Chen C.T., Yeh C.N., Chang Y., Sung H.W. (2019). Phase-Changeable Nanoemulsions for Oral Delivery of a Therapeutic Peptide: Toward Targeting the Pancreas for Antidiabetic Treatments Using Lymphatic Transport. Adv. Funct. Mater..

[67] Harrison G.A. (1923). Insulin in alcoholic solution by the mouth. Br. Med. J..

[68] Sonaje K., Lin Y.-H., Juang J.-H., Wey S.-P., Chen C.-T., Sung H.-W. (2009). In vivo evaluation of safety and efficacy of self-assembled nanoparticles for oral insulin delivery. Biomaterials.

[69] Wu Z.M., Zhou L., Guo X.D., Jiang W., Ling L., Qian Y., Luo K.Q., Zhang L.J. (2012). HP55-coated capsule containing PLGA/RS nanoparticles for oral delivery of insulin. Int. J. Pharm..

[70] Jin Y., Song Y., Zhu X., Zhou D., Chen C., Zhang Z., Huang Y. (2012). Goblet cell-targeting nanoparticles for oral insulin delivery and the influence of mucus on insulin transport. Biomaterials.

[71] Cohn J.N., Archibald D.G., Ziesche S., Franciosa J.A., Harston W.E., Tristani F.E., Dunkman W.B., Jacobs W., Francis G.S., Flohr K.H. (1986). Effect of vasodilator therapy on mortality in chronic congestive heart failure. Results of a Veterans Administration Cooperative Study. N. Engl. J. Med..

[72] MacDougall A.I., Addis G.J., MacKay N., Dymock I.W., Turpie A.G., Ballingall D.L., MacLennan W.J., Whiting B., MacArthur J.G. (1970). Treatment of hypertension with clonidine. Br. Med. J..

[73] Mah G.T., Tejani A.M., Musini V.M. (2009). Methyldopa for primary hypertension. Cochrane Database Syst. Rev..

[74] Bangalore S., Steg G., Deedwania P., Crowley K., Eagle K.A., Goto S., Ohman E.M., Cannon C.P., Smith S.C., Zeymer U. (2012). β-Blocker use and clinical outcomes in stable outpatients with and without coronary artery disease. JAMA.

[75] Lazar H.L. (2005). Role of angiotensin-converting enzyme inhibitors in the coronary artery bypass patient. Ann. Thorac. Surg..

[76] Güleç S. (2014). Valsartan after myocardial infarction. Anadolu Kardiyol. Derg..

[77] Hubers S.A., Brown N.J. (2016). Combined Angiotensin Receptor Antagonism and Neprilysin Inhibition. Circulation.

[78] Fares H., DiNicolantonio J.J., O’Keefe J.H., Lavie C.J. (2016). Amlodipine in hypertension: A first-line agent with efficacy for improving blood pressure and patient outcomes. Open Heart.

[79] Rodríguez Padial L., Barón-Esquivias G., Hernández Madrid A., Marzal Martín D., Pallarés-Carratalá V., de la Sierra A. (2016). Clinical Experience with Diltiazem in the Treatment of Cardiovascular Diseases. Cardiol. Ther..

[80] Badu-Boateng C., Jennings R., Hammersley D. (2018). The therapeutic role of ivabradine in heart failure. Ther. Adv. Chronic Dis..

[81] Pitt B., Remme W., Zannad F., Neaton J., Martinez F., Roniker B., Bittman R., Hurley S., Kleiman J., Gatlin M. (2003). Eplerenone, a selective aldosterone blocker, in patients with left ventricular dysfunction after myocardial infarction. N. Engl. J. Med..

[82] Pitt B., Zannad F., Remme W.J., Cody R., Castaigne A., Perez A., Palensky J., Wittes J. (1999). The Effect of Spironolactone on Morbidity and Mortality in Patients with Severe Heart Failure. N. Engl. J. Med..

[83] Campbell T.J., MacDonald P.S. (2003). Digoxin in heart failure and cardiac arrhythmias. Med. J. Aust..

[84] Ramkumar S., Raghunath A., Raghunath S. (2016). Statin Therapy: Review of Safety and Potential Side Effects. Acta Cardiol. Sin..

[85] Jneid H., Bhatt D.L., Corti R., Badimon J.J., Fuster V., Francis G.S. (2003). Aspirin and clopidogrel in acute coronary syndromes: Therapeutic insights from the CURE study. Arch. Intern. Med..

[86] Tran H., Mehta S.R., Eikelboom J.W. (2006). Clinical update on the therapeutic use of clopidogrel: Treatment of acute ST-segment elevation myocardial infarction (STEMI). Vasc. Health Risk Manag..

[87] Welsh R.C., Sidhu R.S., Cairns J.A., Lavi S., Kedev S., Moreno R., Cantor W.J., Stankovic G., Meeks B., Yuan F. (2019). Outcomes Among Clopidogrel, Prasugrel, and Ticagrelor in ST-Elevation Myocardial Infarction Patients Who Underwent Primary Percutaneous Coronary Intervention From the TOTAL Trial. Can. J. Cardiol..

[88] Date A.A., Desai N., Dixit R., Nagarsenker M. (2010). Self-nanoemulsifying drug delivery systems: Formulation insights, applications and advances. Nanomedicine.

[89] Sun M., Zhai X., Xue K., Hu L., Yang X., Li G., Si L. (2011). Intestinal absorption and intestinal lymphatic transport of sirolimus from self-microemulsifying drug delivery systems assessed using the single-pass intestinal perfusion (SPIP) technique and a chylomicron flow blocking approach: Linear correlation with oral bioavailabilities in rats. Eur. J. Pharm. Sci..

[90] Nekkanti V., Wang Z., Betageri G.V. (2016). Pharmacokinetic evaluation of improved oral bioavailability of valsartan: Proliposomes versus self-nanoemulsifying drug delivery system. AAPS PharmSciTech.

[91] Shafiq S., Shakeel F., Talegaonkar S., Ahmad F.J., Khar R.K., Ali M. (2007). Development and bioavailability assessment of ramipril nanoemulsion formulation. Eur. J. Pharm. Biopharm..

[92] Chhabra G., Chuttani K., Mishra A.K., Pathak K. (2011). Design and development of nanoemulsion drug delivery system of amlodipine besilate for improvement of oral bioavailability. Drug Dev. Ind. Pharm..

[93] Shah U., Joshi G., Sawant K.J. (2014). Improvement in antihypertensive and antianginal effects of felodipine by enhanced absorption from PLGA nanoparticles optimized by factorial design. Mater. Sci. Eng. C.

[94] Dudhipala N., Veerabrahma K. (2015). Pharmacokinetic and pharmacodynamic studies of nisoldipine-loaded solid lipid nanoparticles developed by central composite design. Drug Dev. Ind. Pharm..

[95] Ranpise N.S., Korabu S.S., Ghodake V.N. (2014). Second generation lipid nanoparticles (NLC) as an oral drug carrier for delivery of lercanidipine hydrochloride. Colloids Surf. B Biointerfaces.

[96] Deshpande P.B., Gurram A.K., Deshpande A., Shavi G.V., Musmade P., Arumugam K., Averineni R.K., Mutalik S., Reddy M.S., Udupa N. (2016). A novel nanoproliposomes of lercanidipine: Development, in vitro and preclinical studies to support its effectiveness in hypertension therapy. Life Sci..

[97] Kim Y.I., Fluckiger L., Hoffman M., Lartaud-Idjouadiene I., Atkinson J., Maincent T. (1997). The antihypertensive effect of orally administered nifedipine-loaded nanoparticles in spontaneously hypertensive rats. Br. J. Pharmacol..

[98] Nelson R.H. (2013). Hyperlipidemia as a risk factor for cardiovascular disease. Prim. Care Clin. Off. Pract..

[99] Vavlukis M., Vavlukis A. (2018). Adding ezetimibe to statin therapy: Latest evidence and clinical implications. Drugs Context.

[100] Tziomalos K., Athyros V.G. (2006). Fenofibrate: A novel formulation (Triglide) in the treatment of lipid disorders: A review. Int. J. Nanomed..

[101] Shaker M.A., Elbadawy H.M., Al Thagfan S.S., Shaker M.A. (2021). Enhancement of atorvastatin oral bioavailability via encapsulation in polymeric nanoparticles. Int. J. Pharm..

[102] Sharma M., Mehta I. (2019). Surface stabilized atorvastatin nanocrystals with improved bioavailability, safety and antihyperlipidemic potential. Sci. Rep..

[103] Kumar N., Chaurasia S., Patel R.R., Khan G., Kumar V., Mishra B. (2016). Atorvastatin calcium loaded PCL nanoparticles: Development, optimization, in vitro and in vivo assessments. RSC Adv..

[104] Elmowafy M., Ibrahim H.M., Ahmed M.A., Shalaby K., Salama A., Hefesha H. (2017). Atorvastatin-loaded nanostructured lipid carriers (NLCs): Strategy to overcome oral delivery drawbacks. Drug Deliv..

[105] Jain K., Kumar R.S., Sood S., Gowthamarajan K. (2013). Enhanced oral bioavailability of atorvastatin via oil-in-water nanoemulsion using aqueous titration method. J. Pharm. Sci. Res..

[106] Tiwari R., Pathak K. (2011). Nanostructured lipid carrier versus solid lipid nanoparticles of simvastatin: Comparative analysis of characteristics, pharmacokinetics and tissue uptake. Int. J. Pharm..

[107] Dudhipala N., Veerabrahma K. (2017). Improved anti-hyperlipidemic activity of Rosuvastatin Calcium via lipid nanoparticles: Pharmacokinetic and pharmacodynamic evaluation. Eur. J. Pharm. Biopharm..

[108] El-Helw A.-R.M., Fahmy U.A. (2015). Improvement of fluvastatin bioavailability by loading on nanostructured lipid carriers. Int. J. Nanomed..

[109] Chen Y., Lu Y., Chen J., Lai J., Sun J., Hu F., Wu W. (2009). Enhanced bioavailability of the poorly water-soluble drug fenofibrate by using liposomes containing a bile salt. Int. J. Pharm..

[110] Mohsin K., Alamri R., Ahmad A., Raish M., Alanazi F.K., Hussain M.D. (2016). Development of self-nanoemulsifying drug delivery systems for the enhancement of solubility and oral bioavailability of fenofibrate, a poorly water-soluble drug. Int. J. Nanomed..

[111] Tran T.H., Ramasamy T., Truong D.H., Choi H.-G., Yong C.S., Kim J.O. (2014). Preparation and characterization of fenofibrate-loaded nanostructured lipid carriers for oral bioavailability enhancement. AAPS Pharmscitech.

[112] Agrawal Y.O., Mahajan U.B., Agnihotri V.V., Nilange M.S., Mahajan H.S., Sharma C., Ojha S., Patil C.R., Goyal S.N. (2021). Ezetimibe-Loaded Nanostructured Lipid Carrier Based Formulation Ameliorates Hyperlipidaemia in an Experimental Model of High Fat Diet. Molecules.

[113] Bandyopadhyay S., Katare O., Singh B. (2012). Optimized self nano-emulsifying systems of ezetimibe with enhanced bioavailability potential using long chain and medium chain triglycerides. Colloids Surf. B Biointerfaces.

[114] Shevalkar G., Vavia P. (2019). Solidified nanostructured lipid carrier (S-NLC) for enhancing the oral bioavailability of ezetimibe. J. Drug Deliv. Sci. Technol..

[115] McLennan D.N., Porter C.J., Charman S.A. (2005). Subcutaneous drug delivery and the role of the lymphatics. Drug Discov. Today Technol..

[116] American Diabetes A. (2020). 2. Classification and Diagnosis of Diabetes: Standards of Medical Care in Diabetes-2020. Diabetes Care.

[117] Hinnen D. (2017). Glucagon-Like Peptide 1 Receptor Agonists for Type 2 Diabetes. Diabetes Spectr..

[118] Roesti E.S., Boyle C.N., Zeman D.T., Sande-Melon M., Storni F., Cabral-Miranda G., Knuth A., Lutz T.A., Vogel M., Bachmann M.F. (2020). Vaccination against amyloidogenic aggregates in pancreatic islets prevents development of type 2 diabetes mellitus. Vaccines.

[119] Zhang Y., Yu X.-L., Zha J., Mao L.-Z., Chai J.-Q., Liu R.-T. (2018). Therapeutic vaccine against IL-1β improved glucose control in a mouse model of type 2 diabetes. Life Sci..

[120] Zha J., Chi X.-W., Yu X.-L., Liu X.-M., Liu D.-Q., Zhu J., Ji H., Liu R.-T. (2016). Interleukin-1β-targeted vaccine improves glucose control and β-cell function in a diabetic KK-Ay mouse model. PLoS ONE.

[121] Cavelti-Weder C., Timper K., Seelig E., Keller C., Osranek M., Lässing U., Spohn G., Maurer P., Müller P., Jennings G.T. (2016). Development of an interleukin-1β vaccine in patients with type 2 diabetes. Mol. Ther..

[122] Pang Z., Nakagami H., Osako M.K., Koriyama H., Nakagami F., Tomioka H., Shimamura M., Kurinami H., Takami Y., Morishita R. (2014). Therapeutic vaccine against DPP4 improves glucose metabolism in mice. Proc. Natl. Acad. Sci. USA.

[123] Qiu Z., Chen X., Zhou Y., Lin J., Ding D., Yang S., Chen F., Wang M., Zhu F., Yu X. (2013). Therapeutic vaccines against human and rat renin in spontaneously hypertensive rats. PLoS ONE.

[124] Brown M.J., Coltart J., Gunewardena K., Ritter J.M., Auton T.R., Glover J.F. (2004). Randomized double-blind placebo-controlled study of an angiotensin immunotherapeutic vaccine (PMD3117) in hypertensive subjects. Clin. Sci..

[125] Hong F., Quan W.Y., Pandey R., Yi S., Chi L., Xia L.Z., Yuan M., Ming L.J. (2011). A vaccine for hypertension based on peptide AngI-R: A pilot study. Int. J. Cardiol..

[126] Chen X., Qiu Z., Yang S., Ding D., Chen F., Zhou Y., Wang M., Lin J., Yu X., Zhou Z. (2013). Effectiveness and safety of a therapeutic vaccine against angiotensin II receptor type 1 in hypertensive animals. Hypertension.

[127] Zhu F., Liao Y.H., Li L.D., Cheng M., Wei F., Wei Y.M., Wang M. (2006). Target organ protection from a novel angiotensin II receptor (AT1) vaccine ATR12181 in spontaneously hypertensive rats. Cell. Mol. Immunol..

[128] Tissot A.C., Maurer P., Nussberger J., Sabat R., Pfister T., Ignatenko S., Volk H.-D., Stocker H., Müller P., Jennings G.T. (2008). Effect of immunisation against angiotensin II with CYT006-AngQb on ambulatory blood pressure: A double-blind, randomised, placebo-controlled phase IIa study. Lancet.

[129] Watanabe R., Suzuki J.-I., Wakayama K., Maejima Y., Shimamura M., Koriyama H., Nakagami H., Kumagai H., Ikeda Y., Akazawa H. (2017). A peptide vaccine targeting angiotensin II attenuates the cardiac dysfunction induced by myocardial infarction. Sci. Rep..

[130] Margulis K., Neofytou E.A., Beygui R.E., Zare R.N. (2015). Celecoxib nanoparticles for therapeutic angiogenesis. ACS Nano.

[131] Vignesh S., Sivashanmugam A., Annapoorna M., Janarthanan R., Subramania I., Jayakumar R. (2018). Injectable deferoxamine nanoparticles loaded chitosan-hyaluronic acid coacervate hydrogel for therapeutic angiogenesis. Colloids Surf. B Biointerfaces.

[132] Tomlinson B., Hu M., Zhang Y., Chan P., Liu Z.-M. (2017). Alirocumab for the treatment of hypercholesterolemia. Expert Opin. Biol. Ther..

[133] Kasichayanula S., Grover A., Emery M.G., Gibbs M.A., Somaratne R., Wasserman S.M., Gibbs J.P. (2018). Clinical Pharmacokinetics and Pharmacodynamics of Evolocumab, a PCSK9 Inhibitor. Clin. Pharmacokinet..

[134] Ray K.K., Wright R.S., Kallend D., Koenig W., Leiter L.A., Raal F.J., Bisch J.A., Richardson T., Jaros M., Wijngaard P.L.J. (2020). Two Phase 3 Trials of Inclisiran in Patients with Elevated LDL Cholesterol. N. Engl. J. Med..

[135] Santos R.D., Raal F.J., Catapano A.L., Witztum J.L., Steinhagen-Thiessen E., Tsimikas S. (2015). Mipomersen, an antisense oligonucleotide to apolipoprotein B-100, reduces lipoprotein (a) in various populations with hypercholesterolemia: Results of 4 phase III trials. Arterioscler. Thromb. Vasc. Biol..

[136] Raal F.J., Kallend D., Ray K.K., Turner T., Koenig W., Wright R.S., Wijngaard P.L., Curcio D., Jaros M.J., Leiter L.A. (2020). Inclisiran for the treatment of heterozygous familial hypercholesterolemia. N. Engl. J. Med..

[137] Witztum J.L., Gaudet D., Freedman S.D., Alexander V.J., Digenio A., Williams K.R., Yang Q., Hughes S.G., Geary R.S., Arca M. (2019). Volanesorsen and triglyceride levels in familial chylomicronemia syndrome. N. Engl. J. Med..

[138] Viney N.J., van Capelleveen J.C., Geary R.S., Xia S., Tami J.A., Rosie Z.Y., Marcovina S.M., Hughes S.G., Graham M.J., Crooke R.M. (2016). Antisense oligonucleotides targeting apolipoprotein (a) in people with raised lipoprotein (a): Two randomised, double-blind, placebo-controlled, dose-ranging trials. Lancet.

[139] Pharma I. (2020). Positive Phase 2 Clinical Data of AKCEA-APOCIII-L(Rx) at ESC Congress 2020.

[140] Graham M.J., Lee R.G., Brandt T.A., Tai L.-J., Fu W., Peralta R., Yu R., Hurh E., Paz E., McEvoy B.W. (2017). Cardiovascular and metabolic effects of ANGPTL3 antisense oligonucleotides. N. Engl. J. Med..

[141] Kawakami R., Nozato Y., Nakagami H., Ikeda Y., Shimamura M., Yoshida S., Sun J., Kawano T., Takami Y., Noma T. (2018). Development of vaccine for dyslipidemia targeted to a proprotein convertase subtilisin/kexin type 9 (PCSK9) epitope in mice. PLoS ONE.

[142] Landlinger C., Pouwer M.G., Juno C., van der Hoorn J.W., Pieterman E.J., Jukema J.W., Staffler G., Princen H.M., Galabova G. (2017). The AT04A vaccine against proprotein convertase subtilisin/kexin type 9 reduces total cholesterol, vascular inflammation, and atherosclerosis in APOE* 3Leiden. CETP mice. Eur. Heart J..

[143] Crossey E., Amar M.J., Sampson M., Peabody J., Schiller J.T., Chackerian B., Remaley A.T. (2015). A cholesterol-lowering VLP vaccine that targets PCSK9. Vaccine.

[144] Olearczyk J., Gao S., Eybye M., Yendluri S., Andrews L., Bartz S., Cully D., Tadin-Strapps M. (2014). Targeting of hepatic angiotensinogen using chemically modified siRNAs results in significant and sustained blood pressure lowering in a rat model of hypertension. Hypertens. Res..

[145] Hsu C.-N., Tain Y.-L. (2021). Targeting the Renin–Angiotensin–Aldosterone System to Prevent Hypertension and Kidney Disease of Developmental Origins. Int. J. Mol. Sci..

[146] Ames M.K., Atkins C.E., Pitt B. (2019). The renin-angiotensin-aldosterone system and its suppression. J. Vet. Intern. Med..

[147] U.S. Food and Drug Administration (2013). Kynamro (Mipomersen Sodium) Injection: Drug Approval Package. https://www.accessdata.fda.gov/drugsatfda_docs/nda/2013/203568Orig1s000TOC.cfm.

[148] Fogacci F., Ferri N., Toth P.P., Ruscica M., Corsini A., Cicero A.F. (2019). Efficacy and safety of mipomersen: A systematic review and meta-analysis of randomized clinical trials. Drugs.

[149] Geary R.S., Baker B.F., Crooke S.T. (2015). Clinical and preclinical pharmacokinetics and pharmacodynamics of mipomersen (Kynamro^®^): A second-generation antisense oligonucleotide inhibitor of apolipoprotein B. Clin. Pharmacokinet..

[150] Santos R.D., Duell P.B., East C., Guyton J.R., Moriarty P.M., Chin W., Mittleman R.S. (2015). Long-term efficacy and safety of mipomersen in patients with familial hypercholesterolaemia: 2-year interim results of an open-label extension. Eur. Heart J..

[151] Esan O., Wierzbicki A.S. (2020). Volanesorsen in the Treatment of Familial Chylomicronemia Syndrome or Hypertriglyceridaemia: Design, Development and Place in Therapy. Drug Des. Dev. Ther..

[152] European Medicines Agency (2019). Waylivra (Volanesorsen): Public Assessment Report. https://www.ema.europa.eu/en/medicines/human/EPAR/waylivra.

[153] Gaudet D., Brisson D., Tremblay K., Alexander V.J., Singleton W., Hughes S.G., Geary R.S., Baker B.F., Graham M.J., Crooke R.M. (2014). Targeting APOC3 in the familial chylomicronemia syndrome. N. Engl. J. Med..

[154] European Medicines Agency (2019). Waylivra, INN-Volanesorsen. https://www.ema.europa.eu/en/documents/assessment-report/waylivra-epar-public-assessment-report_en.pdf.

[155] Dyrbuś K., Gąsior M., Penson P., Ray K.K., Banach M. (2020). Inclisiran—New hope in the management of lipid disorders?. J. Clin. Lipidol..

[156] European Medicines Agency (2020). Leqvio (Inclisiran): An Overview of Leqvio and Why It Is Authorised in the EU. https://www.ema.europa.eu/en/medicines/human/EPAR/leqvio.

[157] European Medicines Agency (2020). Leqvio: Assessment Report. https://www.ema.europa.eu/en/documents/assessment-report/leqvio-epar-public-assessment-report_en.pdf.

[158] Fowler A., Sampson M., Remaley A.T., Chackerian B. (2021). A VLP-based vaccine targeting ANGPTL3 lowers plasma triglycerides in mice. bioRxiv.

[159] Brakenhielm E., Alitalo K. (2019). Cardiac lymphatics in health and disease. Nat. Rev. Cardiol..

[160] Ananthakrishnan P., Mariani G., Moresco L., Giuliano A.E. (2008). The anatomy and physiology of lymphatic circulation. Radioguided Surgery.

[161] Supersaxo A., Hein W.R., Steffen H. (1990). Effect of molecular weight on the lymphatic absorption of water-soluble compounds following subcutaneous administration. Pharm. Res..

[162] Hirano K., Hunt C.A. (1985). Lymphatic transport of liposome-encapsulated agents: Effects of liposome size following intraperitoneal administration. J. Pharm. Sci..

[163] Flessner M., Dedrick R., Schultz J.S. (1985). Exchange of macromolecules between peritoneal cavity and plasma. Am. J. Physiol. Heart Circ. Physiol..

[164] Lim H.Y., Thiam C.H., Yeo K.P., Bisoendial R., Hii C.S., McGrath K.C., Tan K.W., Heather A., Alexander J.S.J., Angeli V. (2013). Lymphatic vessels are essential for the removal of cholesterol from peripheral tissues by SR-BI-mediated transport of HDL. Cell Metab..

[165] Reddy S.T., Rehor A., Schmoekel H.G., Hubbell J.A., Swartz M.A. (2006). In vivo targeting of dendritic cells in lymph nodes with poly (propylene sulfide) nanoparticles. J. Control. Release.

[166] Harvey A.J., Kaestner S.A., Sutter D.E., Harvey N.G., Mikszta J.A., Pettis R.J. (2011). Microneedle-based intradermal delivery enables rapid lymphatic uptake and distribution of protein drugs. Pharm. Res..

[167] Hettinga J., Carlisle R. (2020). Vaccination into the Dermal Compartment: Techniques, Challenges, and Prospects. Vaccines.

[168] Moore J.E., Bertram C.D. (2018). Lymphatic System Flows. Annu. Rev. Fluid Mech..

[169] Liao S., Padera T.P. (2013). Lymphatic Function and Immune Regulation in Health and Disease. Lymphat. Res. Biol..

[170] Canzona F., Massimo M., Tuzi A., Maggiori E., Grosso M.G., Antonaci L., Santini S., Catizzone A.R., Troili F., Gallo A. (2020). Intradermal Therapy (mesotherapy) in Dermatology. J. Dermatol. Ski. Sci..

[171] Hawkridge A., Hatherill M., Little F., Goetz M.A., Barker L., Mahomed H., Sadoff J., Hanekom W., Geiter L., Hussey G. (2008). Efficacy of percutaneous versus intradermal BCG in the prevention of tuberculosis in South African infants: Randomised trial. BMJ.

[172] Kroger C.J., Clark M., Ke Q., Tisch R.M. (2018). Therapies to suppress β cell autoimmunity in type 1 diabetes. Front. Immunol..

[173] Clark M., Kroger C.J., Tisch R.M. (2017). Type 1 diabetes: A chronic anti-self-inflammatory response. Front. Immunol..

[174] Richardson S.J., Willcox A., Bone A.J., Morgan N.G., Foulis A.K. (2011). Immunopathology of the human pancreas in type-I diabetes. Seminars in Immunopathology.

[175] Harrison L.C. (2005). The prospect of vaccination to prevent type 1 diabetes. Hum. Vaccines.

[176] Smith E.L., Peakman M. (2018). Peptide immunotherapy for type 1 diabetes—Clinical advances. Front. Immunol..

[177] Thrower S.L., James L., Hall W., Green K.M., Arif S., Allen J.S., Van-Krinks C., Lozanoska-Ochser B., Marquesini L., Brown S. (2009). Proinsulin peptide immunotherapy in type 1 diabetes: Report of a first-in-man Phase I safety study. Clin. Exp. Immunol..

[178] Dul M., Nikolic T., Stefanidou M., McAteer M., Williams P., Mous J., Roep B., Kochba E., Levin Y., Peakman M. (2019). Conjugation of a peptide autoantigen to gold nanoparticles for intradermally administered antigen specific immunotherapy. Int. J. Pharm..

[179] Nikolic T., Zwaginga J.J., Uitbeijerse B.S., Woittiez N.J., de Koning E.J., Aanstoot H.-J., Roep B.O. (2020). Safety and feasibility of intradermal injection with tolerogenic dendritic cells pulsed with proinsulin peptide—For type 1 diabetes. Lancet Diabetes Endocrinol..

[180] Nicoll L.H., Hesby A. (2002). Intramuscular injection: An integrative research review and guideline for evidence-based practice. Appl. Nurs. Res..

[181] Nakajima Y., Mukai K., Takaoka K., Hirose T., Morishita K., Yamamoto T., Yoshida Y., Urai T., Nakatani T. (2017). Establishing a new appropriate intramuscular injection site in the deltoid muscle. Hum. Vaccin. Immunother..

[182] Ogston-Tuck S. (2014). Intramuscular injection technique: An evidence-based approach. Nurs. Stand..

[183] Rodger M.A., King L. (2000). Drawing up and administering intramuscular injections: A review of the literature. J. Adv. Nurs..

[184] Kivelä R., Havas E., Vihko V. (2007). Localisation of lymphatic vessels and vascular endothelial growth factors-C and -D in human and mouse skeletal muscle with immunohistochemistry. Histochem. Cell Biol..

[185] Abai A.M., Hobart P.M., Barnhart K.M. (1999). Insulin delivery with plasmid DNA. Hum. Gene Ther..

[186] Phrommintikul A., Kuanprasert S., Wongcharoen W., Kanjanavanit R., Chaiwarith R., Sukonthasarn A. (2011). Influenza vaccination reduces cardiovascular events in patients with acute coronary syndrome. Eur. Heart J..

[187] Gurfinkel E.P., Mendiz O., Mautner B. (2004). Flu vaccination in acute coronary syndromes and planned percutaneous coronary interventions (FLUVACS) study. Eur. Heart J..

[188] Vlachopoulos C.V., Terentes-Printzios D.G., Aznaouridis K.A., Pietri P.G., Stefanadis C.I. (2015). Association between pneumococcal vaccination and cardiovascular outcomes: A systematic review and meta-analysis of cohort studies. Eur. J. Prev. Cardiol..

[189] Monteiro M.P. (2014). Obesity vaccines. Hum. Vaccines Immunother..

[190] Chawla R., Madhu S., Makkar B., Ghosh S., Saboo B., Kalra S. (2020). RSSDI-ESI clinical practice recommendations for the management of type 2 diabetes mellitus 2020. Int. J. Diabetes Dev. Ctries..

[191] Hulot J.-S., Ishikawa K., Hajjar R.J. (2016). Gene therapy for the treatment of heart failure: Promise postponed. Eur. Heart J..

[192] El-Armouche A., Eschenhagen T. (2009). β-Adrenergic stimulation and myocardial function in the failing heart. Heart Fail. Rev..

[193] Rockman H.A., Chien K.R., Choi D.-J., Iaccarino G., Hunter J.J., Ross J., Lefkowitz R.J., Koch W.J. (1998). Expression of a β-adrenergic receptor kinase 1 inhibitor prevents the development of myocardial failure in gene-targeted mice. Proc. Natl. Acad. Sci. USA.

[194] Harding V.B., Jones L.R., Lefkowitz R.J., Koch W.J., Rockman H.A. (2001). Cardiac βARK1 inhibition prolongs survival and augments β blocker therapy in a mouse model of severe heart failure. Proc. Natl. Acad. Sci. USA.

[195] Shah A.S., White D.C., Emani S., Kypson A.P., Lilly R.E., Wilson K., Glower D.D., Lefkowitz R.J., Koch W.J. (2001). In vivo ventricular gene delivery of a β-adrenergic receptor kinase inhibitor to the failing heart reverses cardiac dysfunction. Circulation.

[196] Rengo G., Lymperopoulos A., Zincarelli C., Donniacuo M., Soltys S., Rabinowitz J.E., Koch W.J. (2009). Clinical Perspective. Circulation.

[197] Tevaearai H.T., Eckhart A.D., Shotwell K.F., Wilson K., Koch W.J. (2001). Ventricular dysfunction after cardioplegic arrest is improved after myocardial gene transfer of a β-adrenergic receptor kinase inhibitor. Circulation.

[198] Raake P.W., Schlegel P., Ksienzyk J., Reinkober J., Barthelmes J., Schinkel S., Pleger S., Mier W., Haberkorn U., Koch W.J. (2013). βARKct cardiac gene therapy ameliorates cardiac function and normalizes the catecholaminergic axis in a clinically relevant large animal heart failure model. Eur. Heart J..

[199] Kawase Y., Ly H.Q., Prunier F., Lebeche D., Shi Y., Jin H., Hadri L., Yoneyama R., Hoshino K., Takewa Y. (2008). Reversal of cardiac dysfunction after long-term expression of SERCA2a by gene transfer in a pre-clinical model of heart failure. J. Am. Coll. Cardiol..

[200] Lyon A.R., Bannister M.L., Collins T., Pearce E., Sepehripour A.H., Dubb S.S., Garcia E., O’Gara P., Liang L., Kohlbrenner E. (2011). SERCA2a gene transfer decreases sarcoplasmic reticulum calcium leak and reduces ventricular arrhythmias in a model of chronic heart failure. Circulation.

[201] Greenberg B., Butler J., Felker G.M., Ponikowski P., Voors A.A., Desai A.S., Barnard D., Bouchard A., Jaski B., Lyon A.R. (2016). Calcium upregulation by percutaneous administration of gene therapy in patients with cardiac disease (CUPID 2): A randomised, multinational, double-blind, placebo-controlled, phase 2b trial. Lancet.

[202] Ylä-Herttuala S. (2012). Endgame: Glybera finally recommended for approval as the first gene therapy drug in the European union. Mol. Ther..

[203] Burnett J.R., Hooper A.J., Hegele R.A. (2017). Familial Lipoprotein Lipase Deficiency.

[204] Rip J., Nierman M.C., Sierts J.A., Petersen W., Den Oever K.V., Raalte D.V., Ross C.J., Hayden M.R., Bakker A.C., Dijkhuizen P. (2005). Gene therapy for lipoprotein lipase deficiency: Working toward clinical application. Hum. Gene Ther..

[205] Bryant L.M., Christopher D.M., Giles A.R., Hinderer C., Rodriguez J.L., Smith J.B., Traxler E.A., Tycko J., Wojno A.P., Wilson J.M. (2013). Lessons learned from the clinical development and market authorization of Glybera. Hum. Gene Ther. Clin. Dev..

[206] Senior M. (2017). After Glybera’s Withdrawal, What’s Next for Gene Therapy?.

[207] Tilemann L., Ishikawa K., Weber T., Hajjar R.J. (2012). Gene therapy for heart failure. Circ. Res..

[208] Huang L.-H., Lavine K.J., Randolph G.J. (2017). Cardiac Lymphatic Vessels, Transport, and Healing of the Infarcted Heart. JACC Basic Transl. Sci..

[209] Hammond H.K., Penny W.F., Traverse J.H., Henry T.D., Watkins M.W., Yancy C.W., Sweis R.N., Adler E.D., Patel A.N., Murray D.R. (2016). Intracoronary gene transfer of adenylyl cyclase 6 in patients with heart failure: A randomized clinical trial. JAMA Cardiol..

[210] Jessup M., Greenberg B., Mancini D., Cappola T., Pauly D.F., Jaski B., Yaroshinsky A., Zsebo K.M., Dittrich H., Hajjar R.J. (2011). Calcium upregulation by percutaneous administration of gene therapy in cardiac disease (CUPID) a phase 2 trial of intracoronary gene therapy of sarcoplasmic reticulum Ca^2+^-ATPase in patients with advanced heart failure. Circulation.

[211] Hulot J.S., Salem J.E., Redheuil A., Collet J.P., Varnous S., Jourdain P., Logeart D., Gandjbakhch E., Bernard C., Hatem S.N. (2017). Effect of intracoronary administration of AAV1/SERCA2a on ventricular remodelling in patients with advanced systolic heart failure: Results from the AGENT-HF randomized phase 2 trial. Eur. J. Heart Fail..

[212] Precigen Triple-Gene (2020). Precigen Triple-Gene Provides Six-Month Follow-Up Data from Phase I Study of INXN-4001, a Multigenic Investigational Therapeutic Candidate for Heart Failure. https://www.prnewswire.com/news-releases/precigen-triple-gene-provides-six-month-follow-up-data-from-phase-i-study-of-inxn-4001-a-multigenic-investigational-therapeutic-candidate-for-heart-failure-301107258.html.

[213] Model M.I. (2010). 425. Arginine and Tetrahydrobiopterin Synergistically Potentiate the Antirestenotic Effect of Vascular Gene Therapy with Inducible Nitric Oxide Synthase. Mol. Ther..

[214] Chung E.S., Miller L., Patel A.N., Anderson R.D., Mendelsohn F.O., Traverse J., Silver K.H., Shin J., Ewald G., Farr M.J. (2015). Changes in ventricular remodelling and clinical status during the year following a single administration of stromal cell-derived factor-1 non-viral gene therapy in chronic ischaemic heart failure patients: The STOP-HF randomized Phase II trial. Eur. Heart J..

[215] Juventas Therapeutics (2014). Juventas Therapeutics Completes Enrollment of Phase I/II RETRO-HF Trial and Demonstrates Safety for Retrograde Infusion of JVS-100 in Patients with Heart Failure. https://www.prnewswire.com/news-releases/juventas-therapeutics-completes-enrollment-of-phase-iii-retro-hf-trial-and-demonstrates-safety-for-retrograde-infusion-of-jvs-100-in-patients-with-heart-failure-270890361.html.

[216] Anttila V., Saraste A., Knuuti J., Jaakkola P., Hedman M., Svedlund S., Lagerström-Fermér M., Kjaer M., Jeppsson A., Gan L.-M. (2020). Synthetic mRNA encoding VEGF-A in patients undergoing coronary artery bypass grafting: Design of a phase 2a clinical trial. Mol. Ther. Methods Clin. Dev..

[217] Gan L.-M., Lagerström-Fermér M., Carlsson L.G., Arfvidsson C., Egnell A.-C., Rudvik A., Kjaer M., Collén A., Thompson J.D., Joyal J. (2019). Intradermal delivery of modified mRNA encoding VEGF-A in patients with type 2 diabetes. Nat. Commun..

[218] Ishikawa K., Fish K.M., Tilemann L., Rapti K., Aguero J., Santos-Gallego C.G., Lee A., Karakikes I., Xie C., Akar F.G. (2014). Cardiac I-1c overexpression with reengineered AAV improves cardiac function in swine ischemic heart failure. Mol. Ther..

[219] Kim J.S., Hwang H., Cho K., Park E., Lee W., Paeng J., Lee D., Kim H., Sohn D., Kim K. (2013). Intramyocardial transfer of hepatocyte growth factor as an adjunct to CABG: Phase I clinical study. Gene Ther..

[220] Hartikainen J., Hassinen I., Hedman A., Kivelä A., Saraste A., Knuuti J., Husso M., Mussalo H., Hedman M., Rissanen T.T. (2017). Adenoviral intramyocardial VEGF-DΔNΔC gene transfer increases myocardial perfusion reserve in refractory angina patients: A phase I/IIa study with 1-year follow-up. Eur. Heart J..

[221] Liu J., Wu P., Wang Y., Du Y. (2016). Ad-HGF improves the cardiac remodeling of rat following myocardial infarction by upregulating autophagy and necroptosis and inhibiting apoptosis. Am. J. Transl. Res..

[222] Wang W., Wang M.-Q., Wang H., Gao W., Zhang Z., Zhao S., Xu H.-Z., Chen B., Zhu M.-X., Wu Z.-Z. (2016). Effects of adenovirus-mediated hepatocyte growth factor gene therapy on postinfarct heart function: Comparison of single and repeated injections. Hum. Gene Ther..

[223] Hardy N., Viola H.M., Johnstone V.P., Clemons T.D., Cserne Szappanos H., Singh R., Smith N.M., Iyer K.S., Hool L.C. (2015). Nanoparticle-mediated dual delivery of an antioxidant and a peptide against the L-Type Ca^2+^ channel enables simultaneous reduction of cardiac ischemia-reperfusion injury. ACS Nano.

[224] Carpentier A.C., Frisch F., Labbe S.M., Gagnon R., de Wal J., Greentree S., Petry H., Twisk J., Brisson D., Gaudet D. (2012). Effect of alipogene tiparvovec (AAV1-LPLS447X) on postprandial chylomicron metabolism in lipoprotein lipase-deficient patients. J. Clin. Endocrinol..

[225] Anselmo A.C., Mitragotri S. (2019). Nanoparticles in the clinic: An update. Bioeng. Transl. Med..

[226] Kobayashi K., Maeda K., Takefuji M., Kikuchi R., Morishita Y., Hirashima M., Murohara T. (2017). Dynamics of angiogenesis in ischemic areas of the infarcted heart. Sci. Rep..

[227] Post M.J., Sato K., Murakami M., Bao J., Tirziu D., Pearlman J.D., Simons M. (2006). Adenoviral PR39 improves blood flow and myocardial function in a pig model of chronic myocardial ischemia by enhancing collateral formation. Am. J. Physiol. -Regul. Integr. Comp. Physiol..

[228] Du C., Chen X.-W., Wang Z.-M., Meng H.-Y., Li Y.-F., Wei T.-W., Wang L.-S. (2020). HGF Treatment Promotes Cardiac Function and Cardiac Repair: Meta-analysis of Pig Models with Myocardial Infarction. Res. Sq..

[229] Korpisalo P., Karvinen H., Rissanen T.T., Kilpijoki J., Marjomäki V., Baluk P., McDonald D.M., Cao Y., Eriksson U., Alitalo K. (2008). VEGF-A and PDGF-B combination gene therapy prolongs angiogenic effects via recruitment of interstitial mononuclear cells and paracrine effects rather than improved pericyte coverage of angiogenic vessels. Circ. Res..

[230] Jin J.-F., Zhu L.-L., Chen M., Xu H.-M., Wang H.-F., Feng X.-Q., Zhu X.-P., Zhou Q. (2015). The optimal choice of medication administration route regarding intravenous, intramuscular, and subcutaneous injection. Patient Prefer. Adherence.

[231] Claassen V. (1994). 2—Intravenous Drug Administration. Techniques in the Behavioral and Neural Sciences.

[232] Xie Y., Bagby T.R., Cohen M.S., Forrest M.L. (2009). Drug delivery to the lymphatic system: Importance in future cancer diagnosis and therapies. Expert Opin. Drug Deliv..

[233] Caliph S.M., Trevaskis N.L., Charman W.N., Porter C.J. (2012). Intravenous dosing conditions may affect systemic clearance for highly lipophilic drugs: Implications for lymphatic transport and absolute bioavailability studies. J. Pharm. Sci..

[234] Yadav P., McLeod V.M., Nowell C.J., Selby L.I., Johnston A.P.R., Kaminskas L.M., Trevaskis N.L. (2018). Distribution of therapeutic proteins into thoracic lymph after intravenous administration is protein size-dependent and primarily occurs within the liver and mesentery. J. Control. Release.

[235] Cabrales P., Han G., Roche C., Nacharaju P., Friedman A.J., Friedman J.M. (2010). Sustained release nitric oxide from long-lived circulating nanoparticles. Free Radic. Biol. Med..

[236] Ruiz-Esparza G.U., Segura-Ibarra V., Cordero-Reyes A.M., Youker K.A., Serda R.E., Cruz-Solbes A.S., Amione-Guerra J., Yokoi K., Kirui D.K., Cara F.E. (2016). A specifically designed nanoconstruct associates, internalizes, traffics in cardiovascular cells, and accumulates in failing myocardium: A new strategy for heart failure diagnostics and therapeutics. Eur. J. Heart Fail..

[237] Marrella A., Iafisco M., Adamiano A., Rossi S., Aiello M., Barandalla-Sobrados M., Carullo P., Miragoli M., Tampieri A., Scaglione S. (2018). A combined low-frequency electromagnetic and fluidic stimulation for a controlled drug release from superparamagnetic calcium phosphate nanoparticles: Potential application for cardiovascular diseases. J. R. Soc. Interface.

[238] Maxwell J.T., Somasuntharam I., Gray W.D., Shen M., Singer J.M., Wang B., Saafir T., Crawford B.H., Jiang R., Murthy N. (2015). Bioactive nanoparticles improve calcium handling in failing cardiac myocytes. Nanomedicine.

[239] Avula U.M.R., Kim G., Lee Y.-E.K., Morady F., Kopelman R., Kalifa J. (2012). Cell-specific nanoplatform-enabled photodynamic therapy for cardiac cells. Heart Rhythm..

[240] Onwordi E.N., Gamal A., Zaman A. (2018). Anticoagulant Therapy for Acute Coronary Syndromes. Interv. Cardiol..

[241] Liu G., Li L., Huo D., Li Y., Wu Y., Zeng L., Cheng P., Xing M., Zeng W., Zhu C. (2017). A VEGF delivery system targeting MI improves angiogenesis and cardiac function based on the tropism of MSCs and layer-by-layer self-assembly. Biomaterials.

[242] Majmudar M.D., Keliher E.J., Heidt T., Leuschner F., Truelove J., Sena B.F., Gorbatov R., Iwamoto Y., Dutta P., Wojtkiewicz G. (2013). Monocyte-directed RNAi targeting CCR2 improves infarct healing in atherosclerosis-prone mice. Circulation.

[243] Ottersbach A., Mykhaylyk O., Heidsieck A., Eberbeck D., Rieck S., Zimmermann K., Breitbach M., Engelbrecht B., Brügmann T., Hesse M. (2018). Improved heart repair upon myocardial infarction: Combination of magnetic nanoparticles and tailored magnets strongly increases engraftment of myocytes. Biomaterials.

[244] Chang M.-Y., Yang Y.-J., Chang C.-H., Tang A.C., Liao W.-Y., Cheng F.-Y., Yeh C.-S., Lai J.J., Stayton P.S., Hsieh P.C. (2013). Functionalized nanoparticles provide early cardioprotection after acute myocardial infarction. J. Control. Release.

[245] Nagaoka K., Matoba T., Mao Y., Nakano Y., Ikeda G., Egusa S., Tokutome M., Nagahama R., Nakano K., Sunagawa K. (2015). A new therapeutic modality for acute myocardial infarction: Nanoparticle-mediated delivery of pitavastatin induces cardioprotection from ischemia-reperfusion injury via activation of PI3K/Akt pathway and anti-inflammation in a rat model. PLoS ONE.

[246] Kassim S.H., Li H., Bell P., Somanathan S., Lagor W., Jacobs F., Billheimer J., Wilson J.M., Rader D.J. (2013). Adeno-associated virus serotype 8 gene therapy leads to significant lowering of plasma cholesterol levels in humanized mouse models of homozygous and heterozygous familial hypercholesterolemia. Hum. Gene Ther..

[247] Greig J.A., Limberis M.P., Bell P., Chen S.-J., Calcedo R., Rader D.J., Wilson J.M. (2017). Nonclinical pharmacology/toxicology study of AAV8. TBG. mLDLR and AAV8. TBG. hLDLR in a mouse model of homozygous familial hypercholesterolemia. Hum. Gene Ther. Clin. Dev..

[248] Fitzgerald K., Frank-Kamenetsky M., Shulga-Morskaya S., Liebow A., Bettencourt B.R., Sutherland J.E., Hutabarat R.M., Clausen V.A., Karsten V., Cehelsky J. (2014). Effect of an RNA interference drug on the synthesis of proprotein convertase subtilisin/kexin type 9 (PCSK9) and the concentration of serum LDL cholesterol in healthy volunteers: A randomised, single-blind, placebo-controlled, phase 1 trial. Lancet.

[249] Nicolau C., Le Pape A., Soriano P., Fargette F., Juhel M.-F. (1983). In vivo expression of rat insulin after intravenous administration of the liposome-entrapped gene for rat insulin I. Proc. Natl. Acad. Sci. USA.

[250] Shifrin A., Auricchio A., Yu Q., Wilson J., Raper S. (2001). Adenoviral vector-mediated insulin gene transfer in the mouse pancreas corrects streptozotocin-induced hyperglycemia. Gene Ther..

[251] Mas A., Montané J., Anguela X.M., Muñoz S., Douar A.M., Riu E., Otaegui P., Bosch F. (2006). Reversal of type 1 diabetes by engineering a glucose sensor in skeletal muscle. Diabetes.

[252] Oh T.K., Li M.Z., Kim S.T. (2006). Gene therapy for diabetes mellitus in rats by intramuscular injection of lentivirus containing insulin gene. Diabetes Res. Clin. Pract..

[253] Muzzin P., Eisensmith R.C., Copeland K.C., Woo S.L. (1997). Hepatic insulin gene expression as treatment for type 1 diabetes mellitus in rats. Mol. Endocrinol..

[254] Thule P., Liu J. (2000). Regulated hepatic insulin gene therapy of STZ-diabetic rats. Gene Ther..

[255] Park Y.M., Woo S., Lee G.T., Ko J.Y., Lee Y., Zhao Z.S., Kim H.J., Ahn C.W., Cha B.S., Kim K.S. (2005). Safety and efficacy of adeno-associated viral vector-mediated insulin gene transfer via portal vein to the livers of streptozotocin-induced diabetic Sprague-Dawley rats. J. Gene Med..

[256] Cheung A.T., Dayanandan B., Lewis J.T., Korbutt G.S., Rajotte R.V., Bryer-Ash M., Boylan M.O., Wolfe M.M., Kieffer T.J. (2000). Glucose-dependent insulin release from genetically engineered K cells. Science.

[257] Encina G., Ezquer F., Conget P., Israel Y. (2011). Insulin is secreted upon glucose stimulation by both gastrointestinal enteroendocrine K-cells and L-cells engineered with the preproinsulin gene. Biol. Res..

[258] Thulé P.M., Campbell A.G., Jia D., Lin Y., You S., Paveglio S., Olson D.E., Kozlowski M. (2015). Long-term glycemic control with hepatic insulin gene therapy in streptozotocin-diabetic mice. J. Gene Med..

[259] Chatenoud L., Primo J., Bach J.-F. (1997). CD3 antibody-induced dominant self tolerance in overtly diabetic NOD mice. J. Immunol..

[260] von Herrath M.G., Coon B., Wolfe T., Chatenoud L. (2002). Nonmitogenic CD3 antibody reverses virally induced (rat insulin promoter-lymphocytic choriomeningitis virus) autoimmune diabetes without impeding viral clearance. J. Immunol..

[261] Herold K.C., Hagopian W., Auger J.A., Poumian-Ruiz E., Taylor L., Donaldson D., Gitelman S.E., Harlan D.M., Xu D., Zivin R.A. (2002). Anti-CD3 monoclonal antibody in new-onset type 1 diabetes mellitus. N. Engl. J. Med..

[262] Herold K.C., Gitelman S.E., Ehlers M.R., Gottlieb P.A., Greenbaum C.J., Hagopian W., Boyle K.D., Keyes-Elstein L., Aggarwal S., Phippard D. (2013). Teplizumab (anti-CD3 mAb) treatment preserves C-peptide responses in patients with new-onset type 1 diabetes in a randomized controlled trial: Metabolic and immunologic features at baseline identify a subgroup of responders. Diabetes.

[263] Herold K.C., Gitelman S.E., Masharani U., Hagopian W., Bisikirska B., Donaldson D., Rother K., Diamond B., Harlan D.M., Bluestone J.A. (2005). A single course of anti-CD3 monoclonal antibody hOKT3γ1 (Ala-Ala) results in improvement in C-peptide responses and clinical parameters for at least 2 years after onset of type 1 diabetes. Diabetes.

[264] Zhang Y.C., Bui J.D., Shen L., Phillips M.I. (2000). Antisense inhibition of β1-adrenergic receptor mRNA in a single dose produces a profound and prolonged reduction in high blood pressure in spontaneously hypertensive rats. Circulation.

[265] Huang H., Li X., Zheng S., Chen Y., Chen C., Wang J., Tong H., Zhou L., Yang J., Zeng C. (2016). Downregulation of Renal G Protein–Coupled Receptor Kinase Type 4 Expression via Ultrasound-Targeted Microbubble Destruction Lowers Blood Pressure in Spontaneously Hypertensive Rats. J. Am. Heart Assoc..

[266] Paulis L., Franke H., Simko F. (2017). Gene therapy for hypertension. Expert Opin. Biol. Ther..

[267] Lázár E., Sadek H.A., Bergmann O. (2017). Cardiomyocyte renewal in the human heart: Insights from the fall-out. Eur. Heart J..

[268] Torchilin V., Klibanov A., Huang L., O’Donnell S., Nossiff N., Khaw B. (1992). Targeted accumulation of polyethylene glycol-coated immunoliposomes in infarcted rabbit myocardium. FASEB J..

[269] Scott R.C., Rosano J.M., Ivanov Z., Wang B., Chong P.L.-G., Issekutz A.C., Crabbe D.L., Kiani M.F. (2009). Targeting VEGF-encapsulated immunoliposomes to MI heart improves vascularity and cardiac function. FASEB J..

[270] Ko Y., Hartner W., Kale A., Torchilin V. (2009). Gene delivery into ischemic myocardium by double-targeted lipoplexes with anti-myosin antibody and TAT peptide. Gene Ther..

[271] Dvir T., Bauer M., Schroeder A., Tsui J.H., Anderson D.G., Langer R., Liao R., Kohane D.S. (2011). Nanoparticles targeting the infarcted heart. Nano Lett..

[272] Pannu H.K., Oliphant M. (2015). The subperitoneal space and peritoneal cavity: Basic concepts. Abdom. Imaging.

[273] Al Shoyaib A., Archie S.R., Karamyan V.T. (2020). Intraperitoneal Route of Drug Administration: Should it Be Used in Experimental Animal Studies?. Pharm. Res..

[274] Michailova K.N., Usunoff K.G. (2006). Serosal Membranes (Pleura, Pericardium, Peritoneum): Normal Structure, Development and Experimental Pathology.

[275] Lee G., Han S., Inocencio I., Cao E., Hong J., Phillips A.R.J., Windsor J.A., Porter C.J.H., Trevaskis N.L. (2019). Lymphatic Uptake of Liposomes after Intraperitoneal Administration Primarily Occurs via the Diaphragmatic Lymphatics and is Dependent on Liposome Surface Properties. Mol. Pharm..

[276] Torres I., Litterst C., Guarino A. (1978). Transport of model compounds across the peritoneal membrane in the rat. Pharmacology.

[277] Turner P.V., Brabb T., Pekow C., Vasbinder M.A. (2011). Administration of substances to laboratory animals: Routes of administration and factors to consider. J. Am. Assoc. Lab. Anim. Sci..

[278] Alberts D.S., Liu P., Hannigan E.V., O’Toole R., Williams S.D., Young J.A., Franklin E.W., Clarke-Pearson D.L., Malviya V.K., DuBeshter B. (1996). Intraperitoneal cisplatin plus intravenous cyclophosphamide versus intravenous cisplatin plus intravenous cyclophosphamide for stage III ovarian cancer. N. Engl. J. Med..

[279] Armstrong D.K., Bundy B., Wenzel L., Huang H.Q., Baergen R., Lele S., Copeland L.J., Walker J.L., Burger R.A. (2006). Intraperitoneal cisplatin and paclitaxel in ovarian cancer. N. Engl. J. Med..

[280] National Cancer Institute (2006). NCI Clinical Announcement on Intraperitoneal Chemotherapy for Ovarian Cancer. https://ctep.cancer.gov/highlights/docs/clin_annc_010506.pdf.

[281] Maranhão R.C., Guido M.C., de Lima A.D., Tavares E.R., Marques A.F., de Melo M.D.T., Nicolau J.C., Salemi V.M., Kalil-Filho R. (2017). Methotrexate carried in lipid core nanoparticles reduces myocardial infarction size and improves cardiac function in rats. Int. J. Nanomed..

[282] Losordo D.W., Vale P.R., Hendel R.C., Milliken C.E., Fortuin F.D., Cummings N., Schatz R.A., Asahara T., Isner J.M., Kuntz R.E. (2002). Phase 1/2 placebo-controlled, double-blind, dose-escalating trial of myocardial vascular endothelial growth factor 2 gene transfer by catheter delivery in patients with chronic myocardial ischemia. Circulation.

[283] Reilly J.P., Grise M.A., Fortuin F.D., Vale P.R., Schaer G.L., Lopez J., Van Camp J.R., Henry T., Richenbacher W.E., Losordo D.W. (2005). Long-term (2-year) clinical events following transthoracic intramyocardial gene transfer of VEGF-2 in no-option patients. J. Interv. Cardiol..

